# MSC‐EVs Prevent Abdominal Aortic Aneurysm Formation by Inhibiting Perivascular Adipose Tissue‐Induced NET Release

**DOI:** 10.1155/sci/8147359

**Published:** 2026-06-10

**Authors:** Xiaowei Sun, Changbo Zhao, Kunfeng Tu, Xun Guan, Lei Lv, Guanhua Xue, Shuofei Yang

**Affiliations:** ^1^ Department of Vascular Surgery, Renji Hospital, School of Medicine, Shanghai Jiao Tong University, Pujian Road 160, Shanghai, 200127, China, sjtu.edu.cn; ^2^ Department of Radiology, Renji Hospital, School of Medicine, Shanghai Jiao Tong University, Pujian Road 160, Shanghai, 200127, China, sjtu.edu.cn

**Keywords:** abdominal aortic aneurysm, neutrophil extracellular trap, perivascular adipose tissue, serum amyloid A

## Abstract

**Background:**

Perivascular adipose tissue (PVAT) envelops arteries and exerts paracrine effects, thereby modulating cardiovascular pathologies. Nevertheless, its precise role in abdominal aortic aneurysm (AAAs) pathogenesis remains elusive. Mesenchymal stem cell‐derived extracellular vesicles (MSC‐EVs) have been implicated in the regulation of neutrophil extracellular traps (NETs) formation. This investigation delineates PVAT’s involvement in AAA, particularly in NETs regulation, and evaluates the modulatory impact of MSC‐EVs on these processes.

**Methods:**

A comprehensive analysis of CT imaging and clinical datasets from AAA patients was undertaken. Subsequent RNA sequencing and transcriptomic profiling of PVAT from AAA animal models and controls were conducted. Both in vivo and in vitro assays were employed to determine whether PVAT‐derived proteins influence NETs release within the abdominal aorta and contribute to AAA development. Additionally, MSC‐EVs were administered via intravenous or intraperitoneal routes to assess their effects on PVAT accumulation and NETs formation.

**Results:**

A significant correlation was established between PVAT characteristics and clinical outcomes in AAA patients. Experimental findings reveal that PVAT‐derived serum amyloid A (SAA) promotes PVAT accumulation and induces NETs release within the abdominal aorta during AAA formation. Intraperitoneal MSC‐EVs administration mitigates AAA development by reducing PVAT accumulation, suppressing SAA expression in PVAT, and inhibiting NETs formation in the abdominal aorta.

**Conclusion:**

PVAT‐derived SAA facilitates NETs formation during AAA pathogenesis. MSC‐EVs, by targeting these mechanisms, offer a promising prophylactic strategy against AAA, opening new therapeutic avenues for future research.

## 1. Introduction

A critical vascular condition where the abdominal aorta shows localized enlargement is called an abdominal aortic aneurysm (AAA). While many aneurysms do not show symptoms, the rupture of an AAA is linked to a mortality risk ranging from 70% to 90% [1]. Despite extensive research spanning decades, there are no known therapies for this condition apart from surgical interventions to replace or reinforce the dilated segment of the aorta [2]. There is a growing need for pharmacological therapy for AAA, and it is of great significance to comprehend the disease’s pathogenesis and mechanisms of AAA formation.

Recent studies indicate that key pathological features of AAA include the infiltration of inflammatory cells into blood vessels and depletion of vascular smooth muscle cells. As a result, the vascular wall gradually thins, and eventually, the structural integrity is compromised. Medical interventions focused on inflammation have shown promise in attenuating AAA progression and reducing the risk of rupture. Our previous studies have identified neutrophil extracellular traps (NETs), which are discharged by neutrophils, as pivotal in the pathogenesis of AAA [3, 4]. Understanding these mechanisms provides insights into potential therapeutic strategies for managing this condition effectively. Neutrophils are activated by internal inflammation and release NETs. Peptidylarginine deiminase 4 (PAD4), encoded by Padi4, is activated at the onset of this complex process, triggering a series of events and producing associated effects.

Perivascular adipose tissue (PVAT) functions as a paracrine mediator that interacts with arteries, playing a pivotal role in the pathophysiology of cardiovascular disorders [5]. PVAT secretes a diverse range of biologically active molecules at various anatomical sites. These molecules, known as adipokines, consist of cytokines, chemokines, and growth factors. A positive association between PVAT volume and aortic dimensions, evident in both thoracic and abdominal areas, has been revealed [6]. Compared to the healthy neck of the aneurysm, increased PVAT density is observed at the site where the AAA diameter is the largest. The impact of PVAT on the disease severity and treatment response in AAA patients remains unclear.

Recent findings indicate that adipocytes in the lesion contribute to the pathogenesis of AAA by inducing interleukin‐18 [7]. It has been demonstrated that the angiotensin II (Ang II) type 1 receptor in PVAT stimulates the occurrence of AAA [8]. PVAT emerged as pivotal in AAA by acting as a reservoir for T lymphocytes and a crucial site for modulating underlying inflammation [9, 10]. However, the mechanisms through which PVAT influences AAA remain unclear. It remains unexplored whether PVAT contributes to AAA formation by regulating NETs. This study investigates the molecular mechanisms involved in PVAT’s role in AAA occurrence and examines the modulatory effects of mesenchymal stem cell‐derived extracellular vesicles (MSC‐EVs) on these mechanisms.

## 2. Materials and Methods

### 2.1. Ethics Approval and Consent to Participate

The study was conducted in accordance with the Declaration of Helsinki. This study received approval from the Institutional Review Board of Renji Hospital, Shanghai Jiao Tong University School of Medicine (Approval Number KY2021‐216, approval date: 1 July 2021, for studies involving animals and tissue and blood samples from patients. Title of the approved project: Study of Contribution of Perivascular Adipose Tissue‐Derived Serum Amyloid A to Abdominal Aortic Aneurysm. Written informed consent for participation in the study and/or the use of samples was obtained from either the patients or their legal representatives.

### 2.2. Human Studies

Individuals with aortoiliac occlusive disease (AIOD) or AAA and healthy controls were among the diverse groups from which participants were recruited. The process’s legitimacy has been confirmed by the approval of the Institutional Review Board at Renji Hospital (Approval Number: KY2021‐216). The information is compiled in Table S1 and includes reliable sources for the following: biochemical and hematological characteristics, clinical outcomes, 1‐year follow‐up data, risk factors, medical history, and demographics. Moreover, patients receiving open abdominal aortic surgery provided PVAT specimens. The Helsinki Declaration’s guidelines were closely followed at every stage of the procedures involving human subjects. Furthermore, written informed consent was obtained prior to sample collection. Organ donors or brain‐dead patients without aneurysms or other cardiovascular disease provided healthy aortic samples. The work has been reported in line with the ARRIVE guidelines 2.0.

### 2.3. AAA Mouse Model Construction and Treatment

All mice used in this study are in the C57BL/6J background. C57BL/6J (WT), TKO (totally deficient in Serum Amyloid A [SAA]1.1, SAA2.1, and SAA3), and TKO‐SAAFAT (exhibiting highly inducible SAA expression only in adipose tissues upon administration of doxycycline) were purchased from the Shanghai Experimental Animal Center and were housed at the Animal Science Center, School of Medicine, Shanghai Jiao Tong University (12‐h/12‐h light/dark cycle at 22°C; two mice per cage). All experiments involving mice were approved by the institutional Ethics Review Committee (Number KY2021–109). All animal experiments were performed in accordance with the Guide for the Care and Use of Laboratory Animals published by the National Institutes of Health (NIH Publication Number 8023, revised 1978). The mice were fed an obesogenic diet for 14 weeks to induce obesity. All mice were given doxycycline (0.4 mg/mL) in drinking water during the past 6 weeks of an obesogenic diet and infused with Ang II (1000 ng/kg per min) during the past 4 weeks (starting 2 weeks after the doxycycline treatment). All animals were anesthetized by inhalation of isoflurane (2%–5%) and euthanized by exsanguination and bilateral thoracotomy after completing the experiments.

Mice were anesthetized by inhalation of isoflurane (induction and maintenance doses were 3%–5% and 1%–2%, respectively). The mice were euthanized by cervical dislocation after all the experiments were done. A mouse model of Ang II‐induced AAA was generated by subcutaneously implanting micro‐osmotic pumps (ALZET DURECT 1004, Durect Corporation, CA, USA) containing saline (isotonic sodium chloride solution) or Ang II (A9525, Sigma‒Aldrich, St. Louis, MO, USA) in 12‐week‐old males. A dose of 1000 ng/kg/min Ang‐II was administered for 28 days, or a drug vehicle was administered intravenously. AAA occurrence was defined as a greater than 50% increase in luminal diameter following 28 days of Ang II infusion.

### 2.4. PVAT Isolation

PVAT was isolated from the AAA model mice after the completion of the modeling protocol. Following the intervention, euthanasia was conducted via the inhalation of an overdose of isoflurane (5%). The abdominal cavity was then opened, and the abdominal aorta was excised, spanning from the diaphragmatic level to the infrarenal bifurcation. The isolated aortic segment was placed in a saline‐filled Petri dish and transferred under a stereomicroscope. Under visual guidance, the periaortic adipose tissue (PVAT) was meticulously dissected away from the aortic wall.

### 2.5. Enhanced Detection of MPO‐DNA Complexes Using Capture ELISA

After coating a 96‐well plate with capture antibody, we used a strategy that involved dilution of 5 μg/mL anti‐MPO mAb (ab9535, Abcam, USA) at 1:500 in 50 μL and then overnight incubation at 4°C. Following three washing cycles, 20 μL of the sample was combined with 80 μL of incubation buffer that contained peroxidase‐labeled anti‐DNA mAb (1:25, Cell Death ELISAPLUS, Roche, Germany), and the outcomes were examined in more detail. After that, the plate was vigorously shaken for 2 h at room temperature (300 rpm). After three additional washing cycles, 100 μL of the peroxidase substrate was added to the wells, and the plate was left to incubate at room temperature in the dark for 20 min. The absorbance was measured at a wavelength of 405 nm. The percentage increase in absorbance compared to that of the control group demonstrated the development of soluble net‐like epitopes.

### 2.6. Enzyme‐Linked Immunosorbent Assay

Tissue concentrations of MPO (ab119605, Abcam) and CitH3 (17939; Cayman Chemical, USA) were determined using the respective ELISA kits according to the manufacturer’s instructions. The concentrations of SAA in serum and PVAT tissue were determined using a SAA human ELISA kit (KHA0011, Invitrogen, CA, USA) or a mouse ELISA kit (KMA0021, Invitrogen, CA, USA).

### 2.7. ROS Concentration

The relative ROS concentration in the cell lysates was measured using the ROS Assay Kit (S0033M; Beyotime) according to the manufacturer’s instructions.

### 2.8. Western Blotting

Cellular and homogenized tissue lysis was carried out using the radioimmunoprecipitation assay buffer, with protein quantification performed utilizing the Omni‐Easy Instant BCA Protein Assay Kit (ZJ102; EpiZyme, China). Then, western blotting procedures were executed following established protocols. Protein separation was achieved using 4%–20% gradient gels. The primary antibodies against CitH3 (1:1000; ab5103), PAD4 (1:1000; ab96758), and SAA (1:1000, ab199030) were purchased from Abcam (Cambridge, MA, USA). The anti‐H3 (1:1000; 4499S) and anti‐GAPDH (1:1000; 5174S), and anti‐β‐actin (1:1000, 4970S) antibodies were purchased from Cell Signaling Technology (MA, USA). Densitometric analysis of the protein bands was performed using ImageJ (v1.47). The experiments were performed in triplicate.

### 2.9. Immunofluorescence Staining

For immunofluorescence staining, serial sections (5 μm) of paraffin‐embedded tissues, primary cultured cells, or frozen sections (8 μm) of abdominal aorta with adherent PVAT were fixed in 4% PFA for 15 min and permeabilized with 0.1% Triton X‐100. The sections were blocked with 1% goat serum at room temperature and incubated with the following primary antibodies overnight at 4°C: anti‐rabbit CitH3 (1:200, ab5103, Abcam, USA), anti‐rabbit MPO (1:200, ab208670, Abcam, USA), and anti‐rabbit Perilipin‐1 (Plin1, 1:200, 15294 1 AP, Proteintech, China). For detection of administered MSC EVs, tissue sections from mice injected with DiD labeled EVs were processed similarly, preserving the intrinsic red fluorescence of the DiD label. For the TdT‐mediated dUTP nick‐end labeling (TUNEL) assay, the sections were incubated with TUNEL reaction buffer (C1086, Beyotime, China) at 37°C for 1 h in the dark. The following day, the sections were incubated with appropriate fluorescently labeled secondary antibodies diluted in blocking buffer at room temperature for 1 h and mounted with 4^′^, 6‐diamidino‐2‐phenylindole (Vector, ZsBio, Beijing, China). Images were captured using a confocal microscope (Leica SP8, Wetzlar, Germany).

### 2.10. RNA Sequencing and Differential Gene Expression Analysis and Gene Ontology (GO) Enrichment Analysis

RNA of the aortic PVAT was extracted using the Trizol reagent. Subsequently, libraries were constructed employing an RNA Sample Prep V2 kit (Illumina, San Diego, USA), and sequencing was performed on an Illumina HiSeq2500 platform. OE Biotechnology Co. Ltd (Shanghai, China) undertook the responsibilities for transcriptome sequencing and analysis. *p*‐Value < 0.05 was established as the criteria for significant differential expression. Hierarchical cluster analysis was then conducted on the differentially expressed genes (DEGs) to elucidate the gene expression patterns across various groups and samples. The analysis of DEGs was conducted using the DESeq2 package (Version 1.32.0) within the R programming environment (Version 4.1.1). For the functional annotation and pathway enrichment of DEGs, GO enrichment analysis was carried out using the Database for Annotation, Visualization, and Integrated Discovery (DAVID). The GO enrichment analysis encompassed three categories: biological processes, cellular components, and molecular functions. And heatmaps illustrating the DEGs and the results of the GO enrichment analysis were generated using the heatmap package (Version 1.0.12) in R. The raw sequencing reads generated in this study have been deposited in the Sequence Read Archive (SRA) of NCBI, Bioproject Accession Number: PRJNA1140895.

### 2.11. Isolation and Characterization of MSC‐EVs

The protocols described in our earlier research were followed in the isolation and characterization of MSC‐EVs [3]. Renji Hospital’s Institutional Review Board approved the MSC‐EVs isolation process. The MSC‐EVs were extracted using human umbilical cord mesenchymal stem cells (hUC‐MSCs), which were provided by the Department of Biotherapy at Renji Hospital. After removing cell debris, the MSC‐EVs were resuspended in PBS and centrifuged at 100,000 g for 2 h at 4°C. The morphological features of MSC‐EVs were assessed using transmission electron microscopy (TEM; JEM‐1200EX, JEOL, Japan), and their size distribution was observed using dynamic light scattering (Litesizer 500, Anton Paar, Austria). Western blot analysis confirmed the presence of EV‐specific biomarkers (CD9, CD63, CD81, and Alix), while calnexin served as a negative control. High sensitivity flow cytometry for nanoparticle analysis was conducted to identify the expression of biomarkers. MSC‐EVs (3 × 10^10^ particles in 100 μL PBS) were injected intraperitoneally and intravenously (tail vein) into animal models at 7, 14, and 21 days after study commencement.

### 2.12. Statistical Analysis

The data are presented as percentages or as the mean ± standard deviation. The normality of the data was assessed using the Kolmogorov–Smirnov test. Before statistical analysis, nonnormally distributed variables underwent log transformation. The Student’s *t*‐test was utilized to compare the continuous variables between the two groups. A one‐way ANOVA was used when comparing more than two groups, and the Student–Newman–Keuls‐q (SNK‐q) post hoc test was used afterward. To compare categorical variables between two or more groups, the chi‐square test was employed. *p*‐Values less than 0.05 were considered statistically significant. SPSS statistical software (v22.0; SPSS, Chicago, IL, USA) was used for data analysis.

## 3. Results

### 3.1. The Quantity and Quality of PVAT Impact Clinical Outcomes in AAA Patients

**Figure 1:**
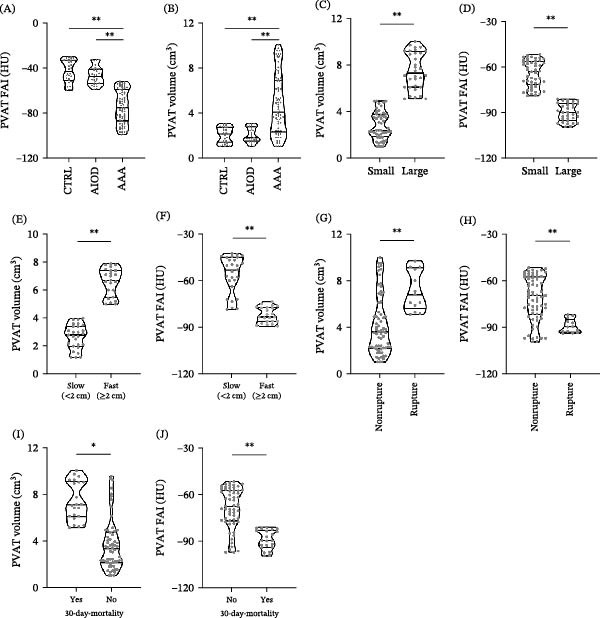
The quantity and quality of PVAT are linked to the clinical outcomes of AAA patients. (A, B) The FAI value of PVAT was markedly lower and the volume of PVAT was markedly larger in patients with AAA than those in patients with aortoiliac occlusive disease (AIOD) and in healthy controls. *N* = 30 in CTRL group, *N* = 30 in AIOD group, *N* = 80 in AAA group, one‐way ANOVA followed by the SNK‐q post hoc test. (C, D) Patients with large‐sized AAAs had a notably larger volume and lower FAI value of PVAT compared to those with small‐sized AAAs. *N* = 48 in small group, *N* = 32 in large group, one‐way ANOVA followed by the SNK‐q post hoc test. (E, F) Patients with rapidly developing AAAs exhibited a significantly larger PVAT volume and lower FAI value than those with slowly developing AAAs. *N* = 27 in slow group, *N* = 23 in fast group, one‐way ANOVA followed by the SNK‐q post hoc test. (G, H) In patients with ruptured AAAs, PVAT volume was considerably larger and FAI values were lower compared to patients with unruptured AAAs. *N* = 67 in nonrupture group, *N* = 13 in rupture group, one‐way ANOVA followed by the SNK‐q post hoc test. (I, J) A low FAI value and substantial PVAT volume were significantly correlated with 30‐day mortality in AAA patients. *N* = 22 in 30‐day‐mortality group, *N* = 58 in non‐30‐day‐mortality group, one‐way ANOVA followed by the SNK‐q post hoc test. For all subfigures:  ^∗^ *p* < 0.05 and  ^∗∗^ *p* < 0.01. Data are presented as mean ± SD.

The amount and quality of PVAT were assessed by computing the volume and fat attenuation index (FAI) of abdominal periaortic fat on CT scan. Both volume and FAI of PVAT emerged as risk factors for 30‐day mortality in AAA patients undergoing surgery, as well as for rapid progression in untreated AAA patients (Table S2). Patients with AAA exhibited significantly lower FAI values and larger PVAT volumes compared to those with AIOD and healthy controls (Figure 1A,B). Additionally, patients with large, ruptured, or rapidly progressing AAA had significantly larger PVAT volume and lower FAI value compared to those with small, unruptured, or slowly progressing AAA (Figure 1C,H). Furthermore, a low FAI value and large PVAT volume exhibited a significant association with 30‐day mortality in AAA patients (Figure 1I,J). CitH3 levels in serum and aortic tissues were higher in AAA patients with larger PVAT volume and lower FAI value (Figure S1A,D). This implies that PVAT might be involved in controlling the formation of NETs.

### 3.2. PVAT‐Derived SAA is Associated With AAA Formation

**Figure 2:**
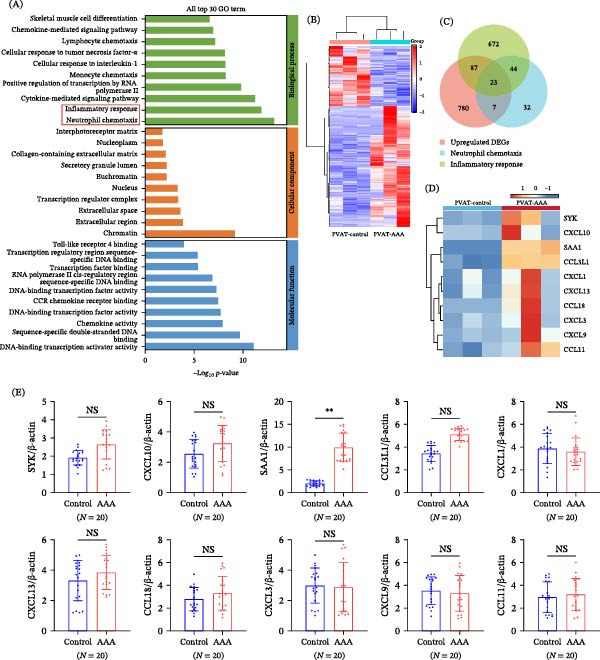
PVAT‐derived SAA is identified as a key molecular of AAA formation by RNA‐seq and transcriptome analysis. (A) The Gene Ontology enrichment analysis indicated that inflammatory response and neutrophil chemotaxis‐associated genes were involved in the main biological process. (B, C) A total of 23 genes were identified according to the Venn diagram obtained from the upregulated differential expressed genes and inflammatory response/neutrophil chemotaxis associated genes. (D) Top 10 upregulated genes among the 23 identified in the Venn diagram. (E) The expression levels of these 10 genes were assessed in PVAT from AAA patients and healthy controls, identifying SAA1 as the target gene. *N* = 20 in control group, *N* = 20 in AAA group, one‐way ANOVA followed by the SNK‐q post hoc test. For all subfigures: NS, not significant;  ^∗∗^ *p* < 0.01. Data are presented as mean ± SD.

We performed RNA‐seq and transcriptome analysis of PVAT from animal models of AAA and controls (Figure 2A,B). A total of 23 genes were identified from the overlap of upregulated DEGs, neutrophil chemotaxis‐related genes, and inflammatory response‐related genes (Figure 2C). The top 10 genes were identified (Figure 2D). PVAT samples from both AAA patients and healthy controls were used to assess the expression of these 10 genes. The results demonstrated elevated expression of PVAT‐derived SAA in both animal models and human AAA patients (Figure 2E).

There is a strong correlation between the clinical outcomes of AAA patients and the levels of SAA in the serum and PVAT. Compared to healthy controls, AAA patients had noticeably higher serum and PVAT SAA levels (Figure S2A,B). Patients with large or ruptured AAAs exhibited significantly higher SAA levels in serum and PVAT than those with small or unruptured AAAs (Figure S2C, F). High SAA levels in both serum and PVAT were significantly linked to 30‐day mortality in AAA patients (Figure S2G, H). Moreover, patients with rapidly advancing AAAs showed significantly higher serum SAA levels compared to those with slowly progressing AAAs, as illustrated in Figure S3I.

AAA patients with a larger volume of PVAT and lower FAI value had higher levels of SAA in serum and PVAT, which suggested expression of SAA was associated with PVAT deposition (Figure S3A,D). The serum level of SAA positively correlated with the serum level of citH3 (Figure S4A). The rate of NETosis in the aortic tissue of AAA patients, as assessed by the quantity of citH3^+^ neutrophils, the percentage of NETosis area in stained sections, and the expression levels of NET markers, is positively associated with the amount of SAA in PVAT. These findings suggest a connection between the formation of AAA and SAA in PVAT (Figure S4B,E).

### 3.3. Adipose Tissue‐Specific SAA Promotes PVAT Deposition and AAA Formation

To get more accurate results, we generated transgenic mice (TKO‐SAA^FAT^) to study the function of adipose‐derived SAA in the development of AAA. When doxycycline was added to the drinking water, we discovered that the mice’s adipose tissue was the only place where SAA was expressed. Furthermore, we found that SAA1.1/2.1 mRNA levels were noticeably higher in the PVAT of TKO‐SAA^FAT^ mice injected with Ang II than in WT mice (Figure S5A). SAA3 mRNA was only detected in WT mice’s PVAT, which is consistent with TKO mice’s lack of endogenous SAA (Figure S5B). Moreover, SAA was not detected in the plasma of TKO or TKO‐SAA^FAT^ mice that received Ang II infusion, but it was considerably higher in the plasma of WT mice that received Ang II infusion (Figure S5C).

**Figure 3:**
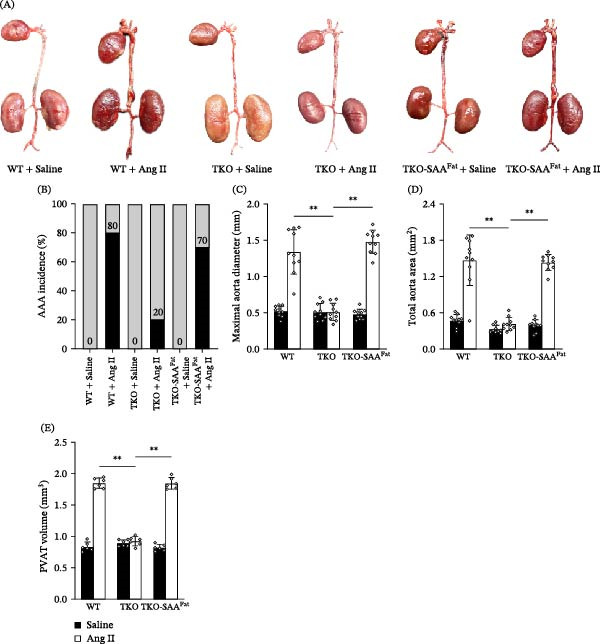
Adipose tissue‐specific SAA promotes deposition of periaortic adipose tissue and AAA formation. (A) Representative macroscopic images of entire aortas harvested from WT, TKO, and TKO‐SAA^FAT^ mice infused with either saline or Ang II. (B) The overall incidence of AAA was significantly lower in TKO mice compared to WT and TKO‐SAA^FAT^ mice. *N* = 10, Student’s *t* test. (C, D) Ang II infusion resulted in a significant increase in the diameter and area of the abdominal aorta in WT and TKO‐SAA^FAT^ mice, but not in TKO mice. *N* = 10, one‐way ANOVA followed by the SNK‐q post hoc test. (E) Ang II infusion caused a significant increase in PVAT volume in WT and TKO‐SAA^FAT^ mice, whereas TKO mice showed no such increase. *N* = 10, one‐way ANOVA followed by the SNK‐q post hoc test. For all subfigures:  ^∗∗^ *p* < 0.01. Data are presented as mean ± SD.

Representative macroscopic images of whole aortas collected from WT, TKO, and TKO‐SAA^FAT^ mice infused with either saline or Ang II are shown in Figure 3A, clearly illustrating the differences. In the meantime, TKO mice showed a markedly reduced overall incidence of AAA in comparison to WT and TKO‐SAA^FAT^ mice (Figure 3B). In WT and TKO‐SAA^FAT^ mice, Ang II infusion significantly increased the abdominal aortic diameter and area; in TKO mice, no such increase was seen (Figure 3C,D). Our research suggests that absence of SAA prevents AAA formation. Importantly, Ang II‐induced AAA can be restored by expressing SAA exclusively in adipose tissue. Further analysis using micro‐CT revealed that Ang II infusion considerably raised the PVAT volume in WT and TKO‐SAA^FAT^ mice, but not in TKO mice (Figure 3E). These findings highlight the crucial part that adipose‐derived SAA plays in encouraging the formation of AAA in response to Ang II stimulation, suggesting that it may be a useful therapeutic target for the clinical prevention or control of AAA.

### 3.4. PVAT‐Derived SAA Facilitates NETs Formation in Abdominal Aorta During AAA Development

**Figure 4:**
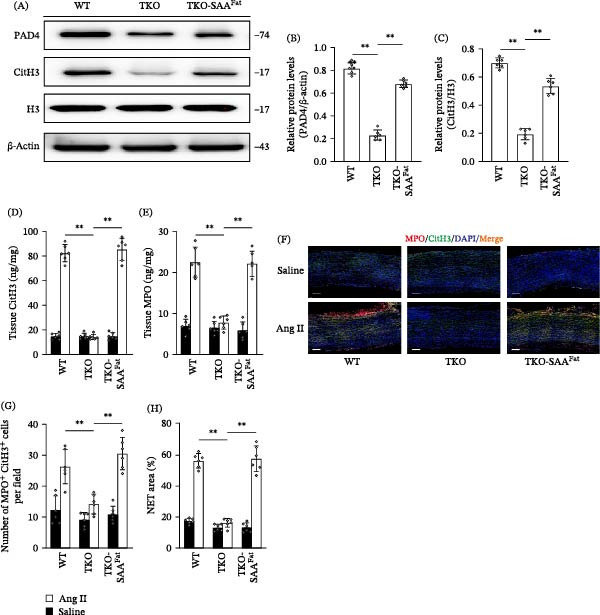
PVAT‐derived SAA promotes NETs formation in the abdominal aorta during AAA formation. (A–C) Expression levels of PAD4 and CitH3 in the abdominal aorta were significantly lower in TKO mice compared to WT and TKO‐SAA^FAT^ mice following Ang II infusion. *N* = 6, one‐way ANOVA followed by the SNK‐q post hoc test. Full‐length blots are presented in Figure S9. (D, E) Significantly elevated levels of CitH3 and MPO were observed in the abdominal aorta of WT and TKO‐SAA^FAT^ mice. *N* = 6, one‐way ANOVA followed by the SNK‐q post hoc test. (F) Immunofluorescence images revealed increased expression of CitH3 and MPO in the aortic media layer of WT and TKO‐SAA^FAT^ mice. Scale bar = 100 μm. (G, H) The number of MPO^+^CitH3^+^ neutrophils and the proportion of neutrophils undergoing NETosis were significantly higher in WT and TKO‐SAA^FAT^ mice, but not in TKO mice, after Ang II infusion. *N* = 6, one‐way ANOVA followed by the SNK‐q post hoc test. For all subfigures:  ^∗∗^ *p* < 0.01. Data are presented as mean ± SD.

In comparison to WT and TKO‐SAA^FAT^ mice, Figure 4A, C demonstrates that TKO mice exhibited significantly lower levels of PAD4 and CitH3 expression, which suggests the formation of NETs in the abdominal aortas after Ang II infusion. Conversely, following Ang II infusion, CitH3 and MPO levels in the abdominal aortas were markedly increased in WT and TKO‐SAA^FAT^ mice but not in TKO mice (Figure 4D,F). In addition, WT and TKO‐SAA^FAT^ mice exhibited notably greater quantities of MPO^+^CitH3^+^ neutrophils and a percentage undergoing NETosis after receiving Ang II infusion compared to TKO mice (Figure 4G,H). Furthermore, by significantly upregulating the expression of PAD4 and CitH3, which was comparable to that of LPS stimulation (Figure S6A,B), SAA‐stimulated neutrophils in vitro increased the percentage of neutrophils undergoing NETosis (Figure S6C,D). Furthermore, MPO‐DNA complexes and reactive oxygen species were released as a result of the stimulation with LPS or SAA (Figure S6E,F).

### 3.5. Regulation of PVAT Deposition and AAA Formation by Intraperitoneal MSC‐EV Injection

**Figure 5:**
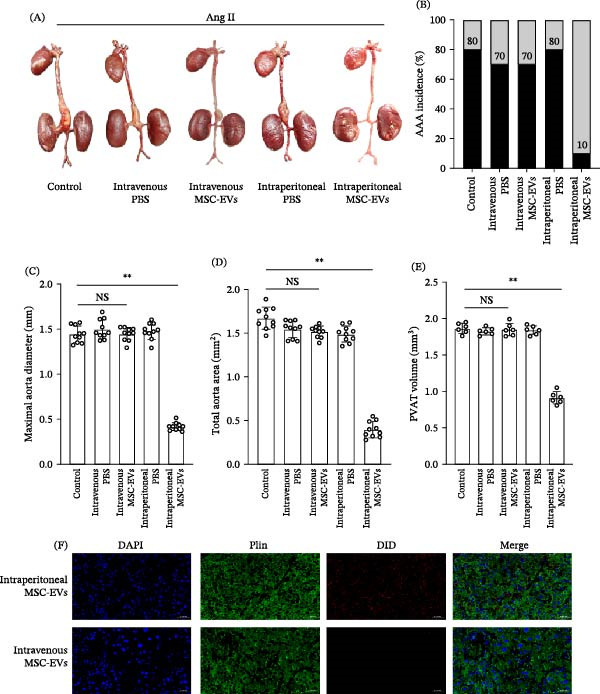
Intraperitoneal injection of MSC‐EVs inhibits the deposition of PVAT and AAA formation. (A) Representative macroscopic images of entire aortas from Ang II‐infused mice receiving either intravenous or intraperitoneal injections of MSC‐EVs. (B) The overall incidence of AAA was significantly lower in mice receiving intraperitoneal MSC‐EVs injections compared to other groups. *N* = 10, Student’s *t* test. (C, D) Intraperitoneal MSC‐EVs injections resulted in a significant reduction in the diameter and area of the abdominal aorta in Ang II‐infused mice. *N* = 10, one‐way ANOVA followed by the SNK‐q post hoc test. (E) Intraperitoneal MSC‐EVs injections significantly inhibited PVAT deposition in WT mice. *N* = 10, one‐way ANOVA followed by the SNK‐q post hoc test. (F) Biodistribution of DiD‐labeled MSC‐EVs to PVAT after intraperitoneal but not intravenous injection, as shown by immunofluorescence. For all subfigures: NS, not significant;  ^∗∗^ *p* < 0.01. Data are presented as mean ± SD.

Sample macroscopic images of entire aortas removed from Ang II‐infused mice receiving intravenous or intraperitoneal injections of MSC‐EVs are shown in Figure 5A. Characterization of the MSC‐EVs is presented in Figure S7A,D. Mice that received intraperitoneal MSC‐EV injections exhibited a significantly lower overall incidence of AAA as compared to other groups (Figure 5B). In mice infused with Ang II, the diameter and area of the abdominal aortas were significantly reduced by intraperitoneally injected MSC‐EVs (Figure 5C,D). Moreover, intraperitoneal MSC‐EV injections significantly reduced PVAT deposition in WT mice (Figure 5E). However, intravenous MSC‐EV injections did not prevent PVAT deposition or AAA formation in mice that had been given Ang II (Figure 5A,E). Importantly, to investigate the mechanistic basis for this route‐dependent efficacy, we performed biodistribution tracing of fluorescently labeled MSC‐EVs. As shown in Figure 5F, intraperitoneally injected MSC‐EVs successfully reached and accumulated within the PVAT, where they colocalized with adipocytes, while intravenously injected MSC‐EVs showed minimal presence in the target tissue. This differential delivery to the pathological niche provides a direct explanation for the specific therapeutic effect observed with the intraperitoneal route.

### 3.6. Intraperitoneal Injection of MSC‐EVs Suppresses SAA Expression in PVAT and Inhibits NET Formation in the Abdominal Aorta

**Figure 6:**
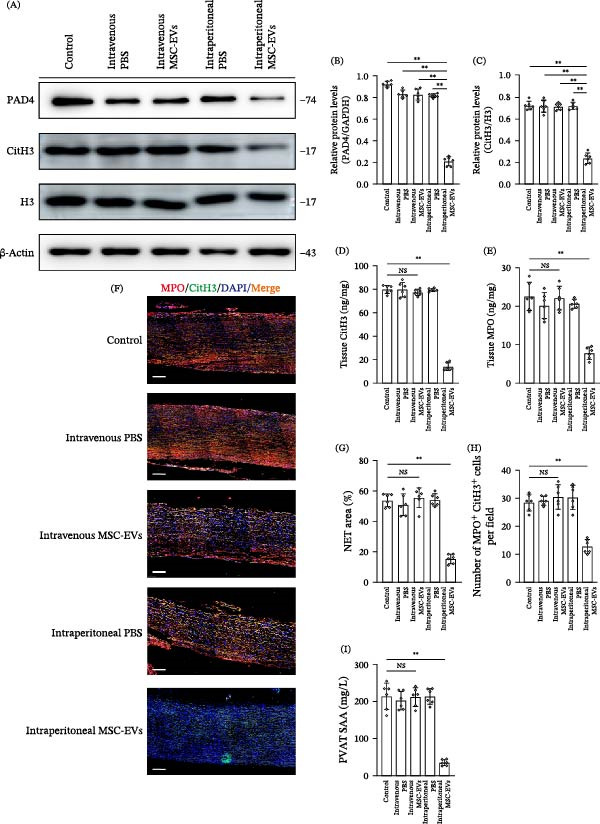
Intraperitoneal injection of MSC‐EVs inhibits SAA expression in PVAT and reduces NET formation in the abdominal aorta. (A–C) Intraperitoneal injection of MSC‐EVs can suppress the expression of PAD4 and CitH3 in the abdominal aorta of mice receiving Ang II infusion. *N* = 6, one‐way ANOVA followed by the SNK‐q post hoc test. Full‐length blots are presented in Figure S10. (D, E) The levels of CitH3 and MPO in the abdominal aorta of Ang II‐infused mice were downregulated following intraperitoneal injection of MSC‐EVs. *N* = 6, one‐way ANOVA followed by the SNK‐q post hoc test. (F) Immunofluorescence images showed that the expression of CitH3 and MPO in the aortic media layer of Ang II‐infused mice was reduced after intraperitoneal injection of MSC‐EVs. Scale bar = 100 μm. (G, H) The number of MPO^+^CitH3^+^ neutrophils and the proportion of neutrophils undergoing NETosis were significantly decreased in Ang II‐infused mice after intraperitoneal injection of MSC‐EVs. *N* = 6, one‐way ANOVA followed by the SNK‐q post hoc test. (I) SAA expression in PVAT in Ang II‐infused mice was suppressed by intraperitoneal injection of MSC‐EVs. *N* = 6, one‐way ANOVA followed by the SNK‐q post hoc test. For all subfigures: NS, not significant;  ^∗∗^ *p* < 0.01. Data are presented as mean ± SD.

MSC‐EVs are injected intraperitoneally to suppress the expression of CitH3 and PAD4 (Figure 6A,C). Our experiments also show that levels of CitH3 and MPO in the abdominal aortas of Ang II‐infused mice are decreased after injection (Figure 6D,F). Furthermore, our study shows a significant decrease in the number of MPO^+^CitH3^+^ neutrophils and the percentage of neutrophils undergoing NETosis in the abdominal aortas of Ang II‐infused mice after treatment with MSC‐EVs (Figure 6G,H). Finally, the injection of MSC‐EVs in the abdominal aortas of Ang II‐infused mice is inhibited (Figure 6I), whereas the expression of SAA in PVAT and the occurrence of NETs in the abdominal aortas of Ang II‐infused mice. To further determine whether the therapeutic effects of intraperitoneally injected MSC‐EVs are dose‐dependent, we divided the mice into low‐, medium‐, and high‐dose MSC‐EV groups. Western blot analysis shows that intraperitoneal injection of MSC‐EVs inhibited SAA expression and NET‐related proteins in a concentration‐dependent manner (Figure S8A,C). Consistently, the levels of CitH3 and MPO decrease in a dose‐dependent manner following MSC‐EV treatment (Figure S8D,F). Furthermore, our study demonstrates that the number of MPO^+^CitH3^+^ neutrophils and the proportion of neutrophils undergoing NETosis in the abdominal aorta of Ang II‐infused mice were reduced in a concentration‐dependent manner after MSC‐EV administration (Figure S8G,H). In addition, the inhibitory effect of intraperitoneally injected MSC‐EVs on SAA expression in PVAT was also dose‐dependent (Figure S7I).

## 4. Discussion

This study discovered a correlation between the clinical outcomes of AAA patients and the characteristics and composition of PVAT. Clinically speaking, most agree that the main indicator of the condition’s advancement is the maximum diameter of AAA [11]. Yet since smaller AAAs may grow more quickly and larger AAAs may grow more slowly, it is useless to forecast rapid growth based solely on maximum diameter measurements. The assessment of extraneous characteristics like intraluminal thrombus and inflammation of the arterial wall has become more crucial in the identification of AAA [12, 13]. The perivascular FAI on CT has been validated as a quantitative measurement indicator for vascular inflammation [14]. Our research indicates that PVAT attenuation can serve as a predictive imaging marker for AAA growth. The notion that inflammation within the local adipose tissue could impact aortic remodeling is substantiated by the discovery that the periaortic adipose tissue attenuation index serves as a significant and independent predictor of the advancement of AAA [15]. The attenuation of perivascular fat on CT angiography was found to be associated with the presence of AAA, and it is suggested that PVAT can promote AAA progression [16, 17].

PVAT could influence the aortic pathophysiology [15]. Examining the inflammatory gene expression in human PVAT associated with AAA may identify novel targets for therapeutic intervention [18]. We identified that PVAT‐derived SAA may mediate the AAA formation. Several studies have suggested a causal relationship between SAA and AAA development in animal models. When mice are given Ang II, for example, the mice produce SAA systemically, but ApoE^−/−^ mice that do not have endogenous acute‐phase SAA are not susceptible to the formation of AAA from Ang II [19]. Additionally, earlier studies have shown that antisense oligonucleotides targeting hepatic SAA can stop Ang II‐induced AAA from developing [20]. While SAA is known to promote Ang II‐induced AAA, its induction is significantly reduced when the myeloperoxidase gene is deleted [21]. It can be deduced that levels of SAA in the blood may signal the nascent phases of AAA formation, possibly prior to the onset of abdominal aortic expansion [22]. The findings of this investigation imply that a significant contributing factor to the asymptomatic onset of vascular diseases is persistent inflammation. However, it is necessary to comprehend the mechanism that causes SAA to become involved to treat these conditions with it. Our experimental findings indicate that SAA derived from PVAT promotes NET formation and PVAT deposition, which ultimately results in the formation of AAA.

Our findings suggest that mesenchymal stem cells may be able to halt the deterioration of aortic elastin, thereby preventing the development of AAA [23]. Nevertheless, it is still unknown what precise processes underlie MSCs’ therapeutic benefits. Extracellular vesicles derived from MSCs have become essential for enabling these therapeutic outcomes [24]. According to a recent study, NET‐induced ferroptosis is inhibited by MSC‐EVs, preventing AAA from developing. The exact effect of MSC‐EVs on AAA is still unknown, though. It has been demonstrated that MSC‐EVs prevent the growth of NETs by giving neutrophils their mitochondria back [25]. By altering the MST4/ERK/Drp1 pathway, exosomal miR‐19b‐3p produced by mesenchymal stem cells can prevent Ang II‐induced AAA and vascular smooth muscle cell senescence [26]. Our data demonstrated that intraperitoneal injection of MSC‐EVs can inhibit the deposition of PVAT and formation of NETs, thereby suppressing AAA formation.

MSC‐EVs have been widely considered a promising cell‐free product in prior research. This is because, despite certain limitations, they maintain the parent cells’ therapeutic effects. MSC‐EVs are not without flaws. Systemic administration of these cells may be less effective due to their quick clearance from target sites. A sustained release strategy of MSC‐EVs into the peritoneal or spinal canal has been proposed as a means of extending their bioavailability at the target sites to expeditiously address this issue. Furthermore, intrathecal injection of MSC‐EVs has demonstrated encouraging results in reducing neuroinflammation and mechanical allodynia related to interstitial cystitis by blocking the activation of the NLRP3 inflammasome [27]. We found that by decreasing SAA expression in PVAT and blocking NET formation in the abdominal aorta, intraperitoneal injection of MSC‐EVs suppresses the formation of AAA. Additionally, cutting‐edge techniques for administering MSC‐EV, like hydrogel‐mediated sustained systemic release, have demonstrated potential for improving hepatic regeneration in individuals suffering from long‐term liver failure [28].

Our research has several limitations. A popular model for studying AAA is the Ang II‐infused mouse model, which is what we used. Our results suggest that further studies should confirm these findings in other AAA models, including the CaCl_2_‐induced and elastase perfusion‐induced models. Second, since our study participants were restricted to people who voluntarily underwent pelvic and abdominal CT scans for medical examinations, conclusions from CT imaging may not be generally applicable. Third, the size of our sample was not very large. Larger‐scale research with longer follow‐up times is therefore required to validate our results. Furthermore, it is still unknown exactly what range of PVAT peripheral organs influence. Our investigation selected a transverse measurement range of 5 mm around the aortic wall based on previously published literature [15]. Fourth, this study did not examine the regulatory mechanisms of SAA derived from PVAT on NET formation. More investigation is required to pinpoint the precise molecular pathways by which SAA drives the AAA progression. Notably, our previous research has demonstrated that MSC‐EVs protect against AAA formation by inhibiting NET‐induced ferroptosis [3]. Integrating these findings with the current data, we speculate that SAA may promote AAA progression at least in part by facilitating NET formation, which in turn triggers ferroptosis in vascular cells. MSC‐EVs, by suppressing the SAA expression in PVAT, could disrupt this cascade, thereby reducing NETosis and subsequent ferroptosis. However, the direct interaction between SAA and NET‐driven ferroptosis remains to be experimentally validated. Elucidating these molecular connections will open the door to new treatment approaches that specifically target factors derived from the adipose tissue in vascular disorders.

## 5. Conclusions

**Figure 7:**
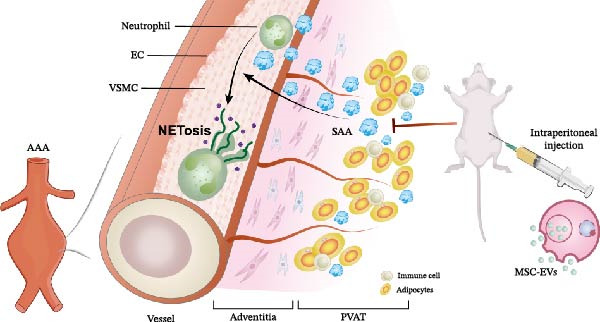
Graphic abstract PVAT‐derived SAA can promote NETs formation in the abdominal aorta during AAA formation. Intraperitoneal injection of MSC‐EVs can suppress SAA expression in PVAT and NET releasing in abdominal aorta to inhibit AAA formation. This research identifies several promising targets for the development of new pharmacological treatments for AAA. AAA, abdominal aortic aneurysm; MSC‐EVs, mesenchymal stem cell‐derived extracellular vesicles; NETs, neutrophil extracellular traps; PVAT, perivascular adipose tissue; SAA, serum amyloid A.

Our study demonstrates that PVAT‐derived SAA promotes NET formation in the abdominal aorta during AAA development. Intraperitoneal injection of MSC‐EVs effectively suppresses SAA expression in PVAT and inhibits NET release, thereby preventing AAA formation (Figure 7). These results point to possible directions for the development of innovative pharmaceutical treatments for AAA; intraperitoneal MSC‐EVs injection is one such treatment strategy.

## Author Contributions

Xiaowei Sun, Lei Lv, Guanhua Xue, and Shuofei Yang drafted the article and contributed to the conception and design and data acquisition and interpretation. Changbo Zhao, Xun Guan, and Kunfeng Tu. contributed to the acquisition and analysis of data.

## Funding

This study was funded by the National Natural Science Foundation of China (Grants 82370497 and 82470494) and the Shanghai Oriental Talent Program Youth Project (Grant No. QNWS2025094).

## Disclosure

The sponsors had no role in the design and conduct of the study, collection, management, analysis, and interpretation of the data, preparation, review, or approval of the manuscript, or decision to submit the manuscript for publication. All named authors meet the International Committee of Medical Journal Editors (ICMJE) criteria for authorship for this article, take responsibility for the integrity of the work as a whole, and have given their approval for this version to be published. All authors read and approved the final manuscript.

## Conflicts of Interest

The authors declare no conflicts of interest.

## Supporting Information

Additional supporting information can be found online in the Supporting Information section.

## Supporting information


**Supporting Information 1** Supporting Information contain supporting methods and supporting results. Supporting results contain Table S1: Demographics and baseline characteristics of healthy controls, AIOD and AAA patients; Table S2: Univariable and multivariable cox regression analysis of risk factors for AAA patients; Figure S1: Characterization of MSC‐EVs; Figure S2: High levels of CitH3 in serum and aortic tissue were associated with low volume and high FAI of PVAT in patients with AAA; Figure S3: Expression of SAA is increased in serum and PVAT in patients with AAA and SAA level in serum and PVAT is associated with clinical outcome of patients with AAA; Figure S4: High SAA in serum and PVAT were associated with low volume and high FAI of PVAT in patients with AAA; Figure S5: SAA level in PVAT positively correlated with NETs formation in abdominal aorta of patients with AAA; Figure S6: TKO‐SAA fat mice exhibited adipose tissue‐specific SAA expression; Figure S7: SAA can promote NETs formation in vitro; Figure S8. Intraperitoneal injection of MSC‐EVs inhibits NET formation and SAA expression in a dose‐dependent manner in the abdominal aorta; original western blot images are compiled in the last part of the supporting figures.

## Data Availability

The data that support the findings of this study are available from the corresponding author upon reasonable request.
