# Supplementary material for: MSC‐EVs Prevent Abdominal Aortic Aneurysm Formation by Inhibiting Perivascular Adipose Tissue‐Induced NET Release
Source: Stem Cells Int. 2026 Jun 10;2026:8147359. doi: 10.1155/sci/8147359 (PMC13250842; doi:10.1155/sci/8147359)
Supplement: Supplementary file 1 — Supporting Information 1 Supporting Information contain supporting methods and supporting results. Supporting results contain Table S1: Demographics and baseline characteristics of healthy controls, AIOD and AAA patients; Table S2: Univariable and multivariable cox regression analysis of risk factors for AAA patients; Figure S1: Characterization of MSC‐EVs; Figure S2: High levels of CitH3 in serum and aortic tissue were associated with low volume and high FAI of PVAT in patients with AAA; Figure S3: Expression of SAA is increased in serum and PVAT in patients with AAA and SAA level in serum and PVAT is associated with clinical outcome of patients with AAA; Figure S4: High SAA in serum and PVAT were associated with low volume and high FAI of PVAT in patients with AAA; Figure S5: SAA level in PVAT positively correlated with NETs formation in abdominal aorta of patients with AAA; Figure S6: TKO‐SAA fat mice exhibited adipose tissue‐specific SAA expression; Figure S7: SAA can promote NETs formation in vitro; Figure S8. Intraperitoneal injection of MSC‐EVs inhibits NET formation and SAA expression in a dose‐dependent manner in the abdominal aorta; original western blot images are compiled in the last part of the supporting figures. [file SCI-2026-8147359-s001.docx]

**Supplementary Materials and Methods**

**Assessment of** **abdominal periaortic** **fat attenuation index (FAI) in AAA patients**

Abdominal CT scans were performed using three different 64-slice CT scanners: the Siemens Healthcare Somatom Sensation 64, the General Electric Discovery CT750 HD, and the Siemens Healthcare Somatom Definition Edge. In the non-enhanced phase, patients are positioned supine with imaging tube voltages ranging from 100 to 140 kV. Additionally, automatic exposure control quality reference milliamperes (QRM) range from 100 to 350 mA can be observed. Images were captured with detector widths ranging from 38.4 mm (64 × 0.6) to 76.8 mm (128 × 0.6) and reconstructed with slice thicknesses of either 3.5 mm or 5 mm, using a field of view of 512 × 512 mm. The section collimation was set at 64 × 0.6 mm, and slice thicknesses were chosen as 3 mm, 3.75 mm, or 5 mm, with increments of 3 mm or 5 mm based on the protocols. The gantry rotation time was set at 50 ms.

To measure the volume of abdominal periaortic (APA) fat, a circular region of interest (ROI) was delineated on each slice, extending 5 mm around the aortic wall. These ROIs were drawn on consecutive slices, covering a region 40 mm above the aortic bifurcation, thus creating a 5 mm cylindrical ROI surrounding the abdominal aortic wall. ^1^ For the standardized measurement of adipose tissue volume, subjects were excluded if the difference between the transverse and anterior-posterior diameter exceeded 5 mm within the volume of interest, preventing the formation of a cylindrical shape. The measurements of APA adipose tissue volume were adjusted based on the anterior-posterior diameter of the aorta and the first slice above the bifurcation. The average fat attenuation within this volume was defined as the APA-FAI.

**MicroCT of mouse abdominal aorta and PVAT**

Mice were anesthetized by inhalation of isoflurane (induction and maintenance doses were 3%–5% and 1%–2%, respectively). Mice were euthanized by cervical dislocation after all the experiments were done. The entire aortas were extracted post-euthanasia from each cohort as delineated previously.^2^ Concisely, the aorta was perfused with saline to eliminate residual blood, using the renal artery and the iliac artery bifurcation as anatomical proximal and distal reference points for abdominal aorta, respectively. Immediately after excision, the aorta was rinsed with phosphate-buffered saline (PBS) and subsequently fixed in formalin for a duration of 24–48 hours on a rocking platform maintained at 4°C. Following three PBS washes, the aortas were immersed in 0.3% phosphotungstic acid, a contrast medium, for 72–96 hours on a rocking platform at 4°C.

Each abdominal aorta was individually imaged in a custom holder with 70% ethanol medium using a Skyscan 1172 (Bruker-Skyscan, Contich, Belgium) microCT system at a voxel resolution of 3 μm, under the following parameters: 40 kVp, 250 µA, 300 ms exposure, 0.40-degree rotation step, and frame averaging of 15. Three-dimensional (3D) reconstructions of the aortas were generated from lateral projections utilizing NRECON (Bruker-Skyscan) software with a beam hardening correction set at 25% and a conebeam reconstruction algorithm. The imaged aortas were analyzed independently. Regions of interest (ROI) were delineated using CT-Analyzer software (Bruker-Skyscan), by tracing the contours of the aortic vascular wall (excluding the tunica adventitia), aortic lumen, and PVAT. Special attention was given to creating ROIs for large veins and their lumens to exclude them from the overall PVAT volume. All ROIs were processed as distinct datasets to determine volumes of interest, specifically aortic PVAT.

For post-scan analysis, we employed direct 3D quantifications, well-documented in bone microarchitecture characterization, such as volume thickness of the aortic wall ^3^. Two independent evaluators (KS and KV) assessed the volume of a random subset of aortas to validate reproducibility. The intraclass correlation coefficients for volume reproducibility were 0.99 and 0.90 for intra- and inter-reader reliability, respectively, indicating high reproducibility for the specified microCT parameters and post-scan tracing protocol. A single reader performed the volume-defining ROIs for this study. For generating 3D renderings, images were imported into a workstation operating in an open VMS environment and processed using Scanco (Scanco Medical, Brüttisellen, Switzerland) 3D morphometry software.

**Assessment of AAA formation in mouse models**

Mice were anesthetized by inhalation of isoflurane (induction dose: 3%–5%). After sedation, anesthesia was maintained using 1% isoflurane during aortic examination, which was performed with an ultrasound device equipped with a 40-MHz transducer (Visual Sonics Vevo 2100, Fujifilm, USA). B-mode ultrasonography was performed to identify the abdominal aorta. Color and spectral Doppler ultrasonography were used to verify whether the direction and velocity of the blood flow were consistent with the aortic signal. Images of the abdominal aortas of the mice were captured, and the maximal aortic diameter was measured as the internal diameter using SPSS Statistics (v22.0; SPSS, Chicago, Illinois, USA). AAA was defined as ≥50% dilatation of the outer diameter of the abdominal aorta compared with the normal outer diameter. Tissue samples were analyzed for pathology and relevant biochemical parameters.

The aorta was observed under a dissecting microscope, and images were captured using a Leica DM6000B microscope (Wetzlar, Germany) after removing the surrounding tissues. The maximal aortic diameter and total aortic area were measured using the inbuilt analysis software provided by Leica (LAS X software, v2017.3.6). Subsequently, the abdominal aorta was harvested, fixed in 4% paraformaldehyde (PFA), and embedded in paraffin.

**Fluorescent Immunostaining: Visualizing Cellular Proteins**

Once being fixed for 15 minutes in 4% PFA, successive 5 μm thick slices from paraffin-embedded tissues or primary cultured cells were permeabilized with 0.1% Triton X-100. The slices were incubated at 4°C with anti-rabbit myeloperoxidase (MPO; 1:200; ab208670, Abcam) and anti-rabbit CitH3 (1:200; ab5103, Abcam) after being blocked for an entire night at room temperature with 1% goat serum. Subsequently, the slices were incubated with fluorescently labeled secondary antibodies (1:200) in blocking buffer for 1 hour at room temperature. 4',6-diamidino-2-phenylindole (Vector, Beijing Vector Science and Technology Co., Ltd.) was used for mounting. A Leica-SP8 confocal microscope in Wetzlar, Germany, was used for all imaging. Two separate investigators counted the average number of double-positive MPO and CitH3 cells in five randomly chosen microscopic fields.

**Quantitative reverse transcription PCR (qRT-PCR)**

Total RNA was isolated from aortic tissues and vascular SMCs using TRIzol (15596018; Invitrogen, Carlsbad, CA, USA), according to the standard protocol. Complementary DNA was obtained from 500 ng of total RNA using a reverse transcription kit (EZBioscience, USA). qRT-PCR was performed using SYBR Green qPCR Master Mix (EZBioscience), and the relative gene expression levels were calculated using the 2^−△△CT^ method. PCR was performed in triplicate in at least three independent experiments, and the results were normalized to the housekeeping gene *β-actin*. The forward (F) and reverse (R) primer sequences for qPCR were as follows:

*SYK*: 5-CATGGAAAAATCTCTCGGGAAGA-3′ (F) and 5′-GTCGATGCGATAGTGCAGCA-3′ (R)

*CXCL10*: 5′-GTGGCATTCAAGGAGTACCTC-3′ (F) and 5′-TGATGGCCTTCGATTCTGGATT-3′ (R)

*SAA1*: 5′-GAGATTCTTTGGCCATGGTGC-3′ (F) and 5′-CCAGCAGGTCGGAAGTGATT-3′ (R)

*CCL3L1*: 5′-CACCTCCCGACAGATTCCAC-3′ (F) and 5′-GGTCACTGACGTATTTCTGGAC-3′ (R)

*CXCL13*, 5′-GCTTGAGGTGTAGATGTGTCC-3′ (F) and 5′- CCCACGGGGCAAGATTTGAA-3′ (R)

*CCL18:* 5′- CTTGTCCTCGTCTGCACCAT-3′ (F) and 5′- CTGGGGGCTGGTTTCAGAAT-3′ (R)

*CXCL3*, 5′-CGCCCAAACCGAAGTCATAG-3′ (F) and 5′- GCTCCCCTTGTTCAGTATCTTTT-3′ (R)

*CXCL9*, 5′-CCAGTAGTGAGAAAGGGTCGC-3′ (F) and 5′- AGGGCTTGGGGCAAATTGTT-3′ (R)

*CCL11*, 5′-CCCCTTCAGCGACTAGAGAG-3′ (F) and 5′- TCTTGGGGTCGGCACAGAT-3′ (R)

**Supplementary Table 1. Demographics and baseline characteristics of healthy controls, AIOD and AAA patients**

| Characteristics | Healthy Controls N=30, % | AIOD patients N=30, % | AAA patients N=80, % | P |
| --- | --- | --- | --- | --- |
| Age, mean ± SD (rang), y | 69.7 ± 12.7 (51-90) | 69.6 ± 11.7 (50-89) | 71.4 ± 10.7 (50-90) | 0.54 |
| Gender, n (%) |  |  |  |  |
| Female | 12 (40.0) | 15 (50.0) | 39 (48.5) | 0.66 |
| Male | 18 (60.0) | 15 (50.0) | 41 (51.3) |  |
| Medical history and risk factors, n (%) |  |  |  |  |
| Smoker status |  |  |  |  |
| Never | 6 (20.0) | 6 (20.0) | 17 (21.3) | 0.15 |
| Past | 8 (26.7) | 9 (30.0) | 23 (28.8) |  |
| Current | 10 (33.3) | 7 (23.3) | 16 (20.0) |  |
| Unknown | 6 (20.0) | 8 (26.7) | 24 (30.0) |  |
| Hypertension | 17 (56.7) | 14 (46.7) | 40 (50.0) | 0.91 |
| Hyperlipidemia | 15 (50.0) | 11 (36.7) | 41 (51.3) | 0.50 |
| Diabetes Mellitus | 18 (60.0) | 17 (56.7) | 44 (55.0) | 0.75 |
| Peripheral artery disease |  |  |  |  |
| No | 15 (50.0) | 13 (43.3) | 50 (62.5) | 0.43 |
| Yes | 15 (50.0) | 17 (56.7) | 30 (37.5) |  |
| Coronary heart disease | 17 (56.7) | 14 (46.7) | 41 (51.3) | 0.98 |
| Stroke | 13 (43.3) | 10 (33.3) | 42 (52.5) | 0.23 |
| COPD | 17 (56.7) | 14 (46.7) | 43 (53.8) | 0.45 |
| Arterial aneurysm family history |  |  |  |  |
| No | 16 (53.3) | 19 (63.3) | 43 (53.8) | <0.05 |
| Yes | 14 (46.7) | 11 (36.7) | 37 (46.3) |  |
| Biochemical and hematological data, mean ± SD |  |  |  |  |
| White blood cells (×10^9^/L) | 7.7 ± 3.1 | 9.7 ± 3.4 | 10.1 ± 3.6 | 0.21 |
| Neutrophils (×10^9^/L) | 3.2 ± 1.8 | 3.8 ± 1.4 | 3.4 ± 1.4 | 0.43 |
| C-reactive protein (mg/L) | 82.5 ± 32.9 | 105.8 ± 27.4 | 92.6 ± 44.2 | 0.22 |
| Creatinine (μmol/L) | 116.0 ± 37.4 | 96.3 ± 29.8 | 112.2 ± 39.6 | 0.68 |
| Blood urea nitrogen (mmol/L) | 7.6 ± 2.4 | 8.3 ± 2.4 | 7.2 ± 3.1 | 0.51 |
| Cholesterol (mg/dL) | 148.5 ± 36.0 | 148.9 ± 29.8 | 154.4 ± 27.9 | 0.52 |
| Triglycerides (mg/dL) | 156.2 ± 28.9 | 153.6 ± 25.8 | 150.4 ± 30.3 | 0.32 |
| ALT, U/L | 31.7 ± 13.4 | 28.8 ± 13.3 | 32.7 ± 12.1 | 0.75 |
| AST, U/L | 28.9 ± 11.8 | 29.0 ± 12.8 | 29.4 ± 11.2 | 0.42 |
| Glucose, mg/dL | 152.4 ± 40.5 | 146.7 ± 38.1 | 156.8 ± 51.1 | 0.62 |
| D-dimer, ng/ml | 100.1 ± 25.5 | 103.8 ± 24.5 | 101.7 ± 29.9 | 0.80 |

AIOD, aortoiliac occlusive disease; AAA, abdominal aortic aneurysm; COPD, chronic obstructive pulmonary disease; SD, standard deviation; ALT, alanine aminotransferase; AST, aspartate transaminase

**Supplementary Table 2.** **Univariable and multivariable cox regression analysis of risk factors for AAA patients**

| Variables | Risk factor for 30d mortality | | | | Risk factor for rapid progression | | | |
| --- | --- | --- | --- | --- | --- | --- | --- | --- |
|  | **AAA patients receiving operation (n = 80)** | | | | **AAA patients under Surveillance (n = 50)** | | | |
|  | **Univariable analysis** | | **Multivariable analysis** | | **Univariable analysis** | | **Multivariable analysis** | |
|  | **HR (95% Cl)** | **P** | **HR (95% Cl)** | **P** | **HR (95% Cl)** | **P** | **HR (95% Cl)** | **P** |
| Age, yr | 1.03 (0.79-1.24) | 0.523 |  |  | 2.23 (1.79-2.71) | 0.734 |  |  |
| Gender (male) | 2.19 (0.68-3.17) | 0.158 |  |  | 1.79 (1.56-2.15) | 0.524 |  |  |
| Hypertension | 0.99 (0.56-1.68) | 0.763 |  |  | 3.19 (2.66-3.42) | 0.395 |  |  |
| Diabetes | 0.69 (0.42-1.89) | 0.571 |  |  | 1.76 (1.51-2.43) | 0.198 |  |  |
| Hyperlipidemia | 2.07 (1.23-3.14) | 0.393 |  |  | 1.77 (1.41-2.13) | 0.265 |  |  |
| Coronary heart disease | 1.39 (0.59-1.47) | 0.672 |  |  | 3.39 (2.39-4.12) | 0.316 |  |  |
| Smoking history | 1.35 (0.48-1.94) | 0.836 |  |  | 1.35 (0.48-1.94) | 0.247 |  |  |
| Maximal diameter, mm | 1.01 (0.93-1.13) | 0.002 | 0.93 (0.90-1.03) | 0.106 | 2.17 (1.95-2.43) | 0.199 |  |  |
| ILT | 1.76 (0.56-2.04) | 0.321 |  |  | 1.13 (0.83-1.96) | 0.621 |  |  |
| PVAT FAI, HU | 1.18 (1.09-1.29) | 0.005 | 1.02 (0.88-1.21) | 0.032 | 1.61 (1.39-1.98) | 0.016 | 1.01 (0.89-1.26) | 0.016 |
| PVAT volume, cm | 2.09 (1.89-2.33) | 0.016 | 1.93 (1.75-2.01) | 0.045 | 2.16 (1.98-3.01) | 0.002 | 1.19 (1.01-1.43) | 0.012 |
| Serum citH3 level, ng/ml | 1.15 (0.99-1.48) | 0.107 |  |  | 2.23 (1.66-2.01) | 0.186 |  |  |

AAA = abdominal aortic aneurysm, ILT = intraluminal thrombus, HR = hazard ratio, CI = confidence interval, PVAT = perivascular adipose tissue, FAI = fat attenuation index, citH3 = citrullinated H3, SAA = Serum Amyloid A

**
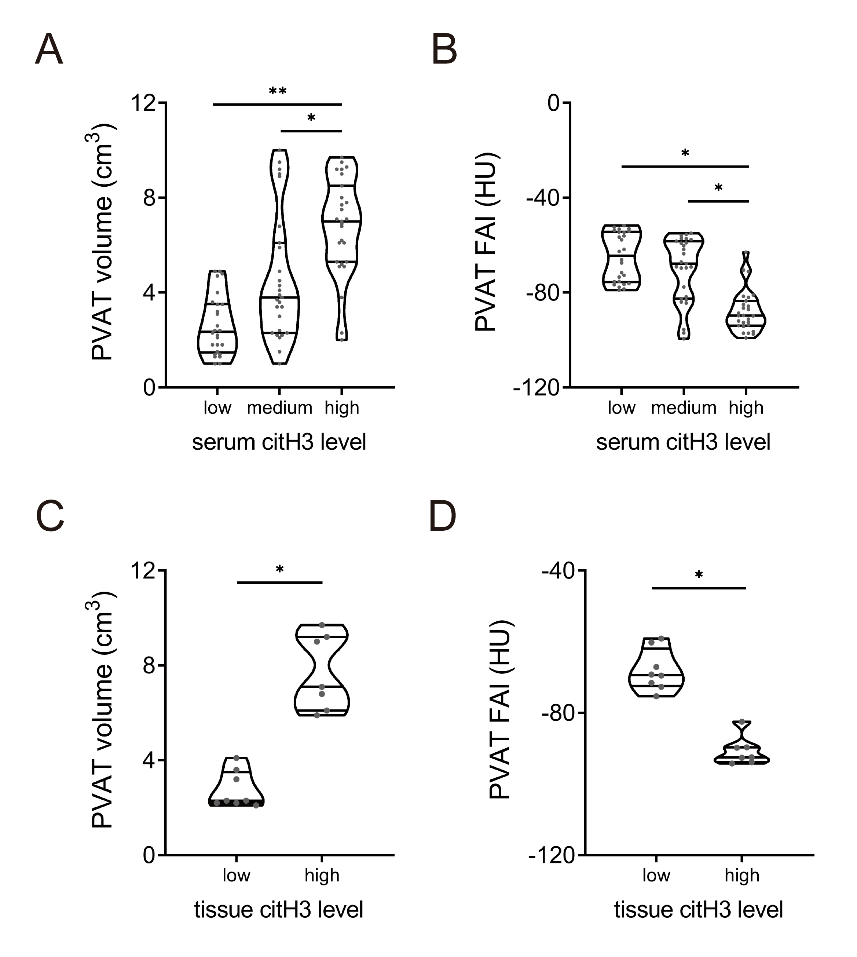
Supplementary Figure 1. High levels of citH3 in serum and aortic tissue were associated with** **low volume and high FAI of PVAT in patients with AAA** A-B. AAA patients with lower FAI value and larger volume of PVAT had higher level of citH3 in serum; C-D. AAA patients with lower FAI value and larger volume of PVAT had higher level of citH3 in aortic tissue. For all subfigures: * *P* < 0.05, ** *P* < 0.01, the data are given as the mean ± SD. One-way ANOVA followed by the SNK-q post hoc test was used.

**
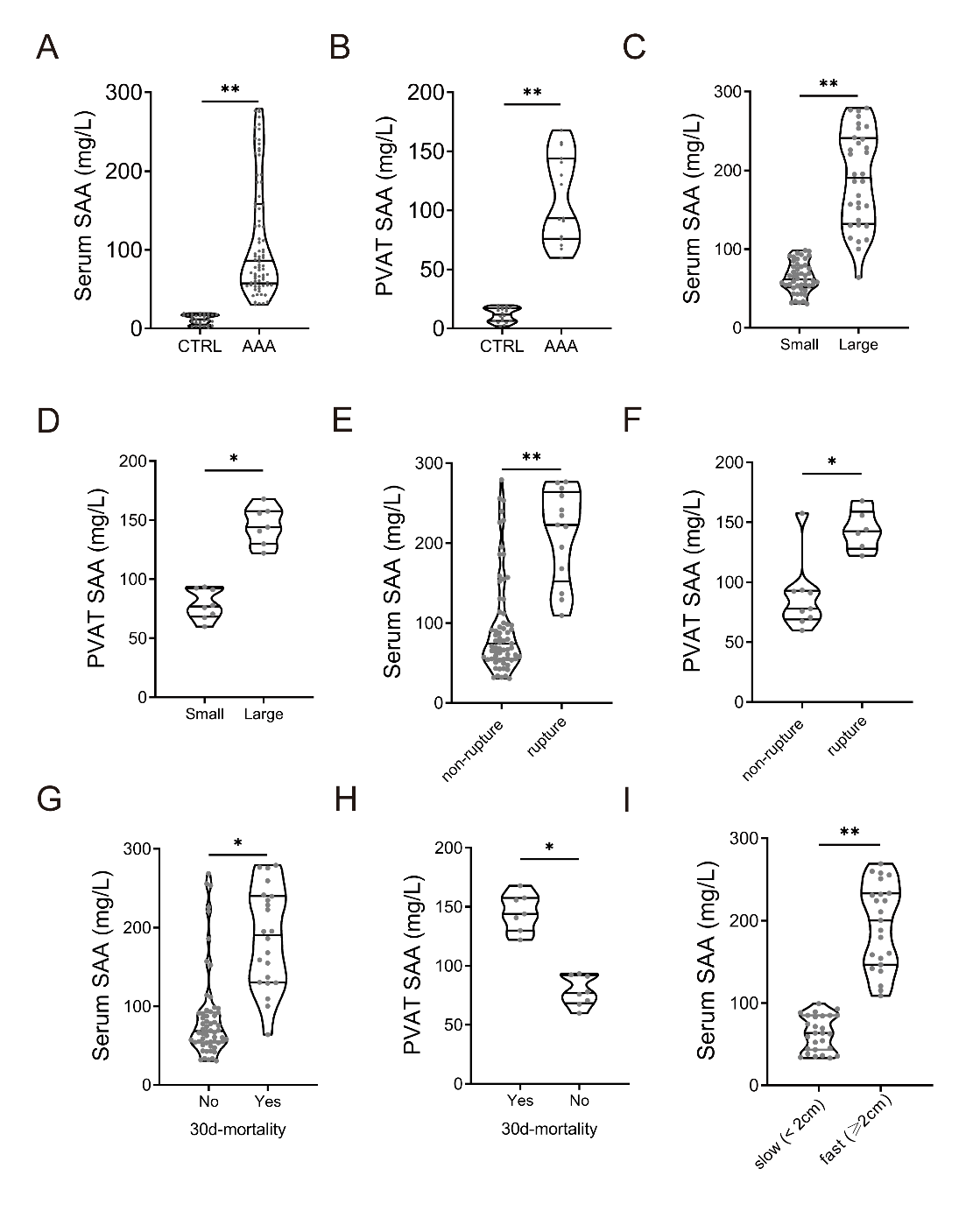
Supplementary Figure 2. Expression of SAA is increased in serum and PVAT in patients with AAA and SAA level in serum and PVAT is** **associated with clinical outcome of patients with AAA** A-B. the levels of SAA in serum and PVAT were significantly higher in patients with AAA than those in healthy controls; C-F. significantly higher levels of SAA in serum and PVAT were observed in patients with large-sized or ruptured AAA than in patients with small-sized or unruptured AAA; G-H. high levels of SAA in serum and PVAT were significantly associated with 30-day mortality in patients with AAA; I. significantly higher level of serum SAA was found in patients with rapidly developing AAA than in patients with slowly developing AAA. For all subfigures: * *P* < 0.05, ** *P* < 0.01, the data are given as the mean ± SD. One-way ANOVA followed by the SNK-q post hoc test was used.

**
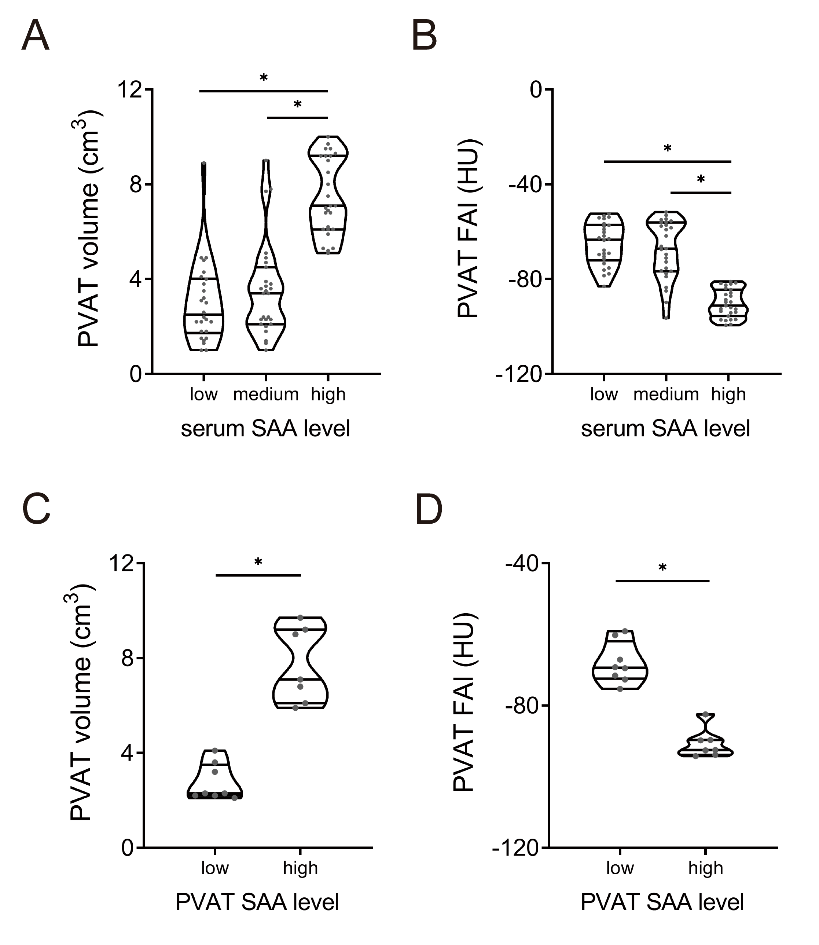
Supplementary Figure 3.** **High SAA in serum and PVAT were associated with** **low volume and high FAI of PVAT in patients with AAA** A-B. AAA patients with larger volume of PVAT and lower FAI value had higher level of SAA in serum; C-D. AAA patients with larger volume of PVAT and lower FAI value had higher level of SAA in PVAT. For all subfigures: * *P* < 0.05, the data are given as the mean ± SD. One-way ANOVA followed by the SNK-q post hoc test was used.

**
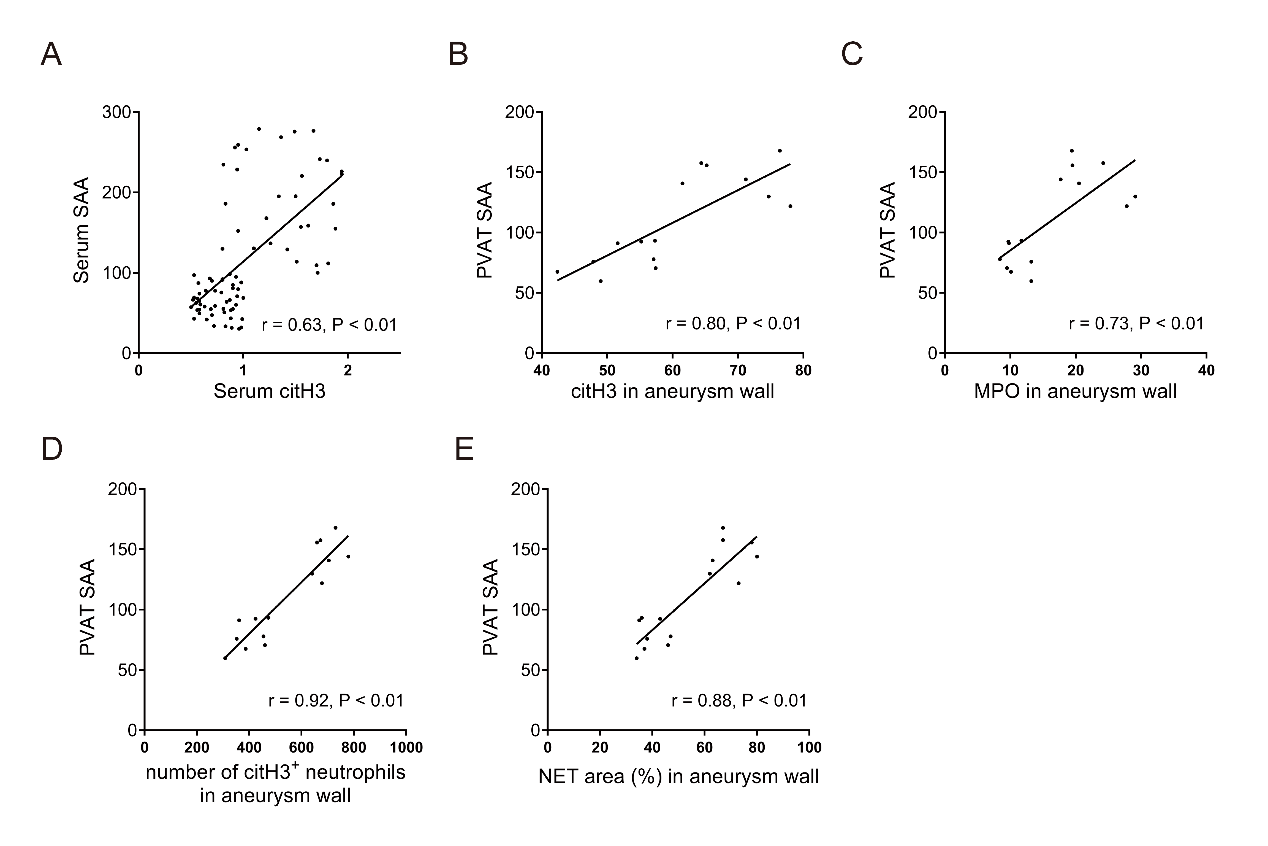
Supplementary Figure 4. SAA level** **in PVAT positively correlated with NETs formation in abdominal aorta of patients with AAA** A. serum level of SAA positively correlated with serum level of citH3; B-C. the level of SAA in PVAT positively correlates with expression level of NET markers (citH3, MPO) in aortic tissue of patients with AAA; D-E. the level of SAA in PVAT positively correlates with the rate of NETosis (number of citH3+ neutrophils, percent of NETosis area in stained section) in aortic tissue of patients with AAA.

**
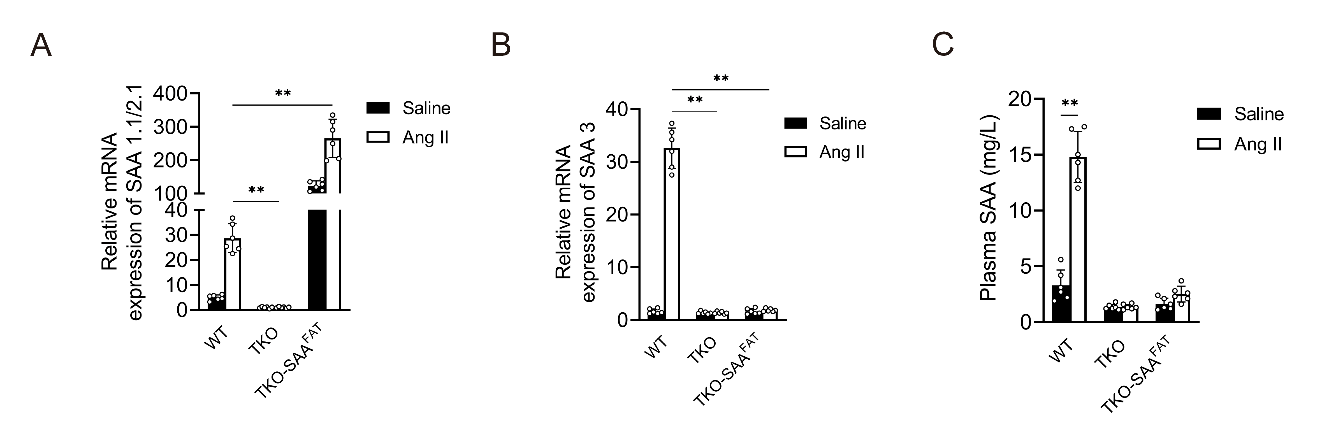
Supplementary Figure 5. TKO-SAA^fat^ mice exhibited** **adipose tissue-specific SAA expression** A. in Ang II infused mice, SAA1.1/2.1 mRNA were significantly higher in PVAT of TKO-SAA^FAT^ mice compared with WT mice; B. consistent with knockout of endogenous SAAs in TKO mice, SAA3 mRNA was only detected in PVAT from WT mice; C. plasma SAA levels elevated significantly in Ang II infused WT mice. SAA was not detected in the plasma of TKO and TKO-SAA^FAT^ mice. For all subfigures: ** *P* < 0.01, NS, non-significant, the data are given as the mean ± SD. One-way ANOVA followed by the SNK-q post hoc test was used.

**
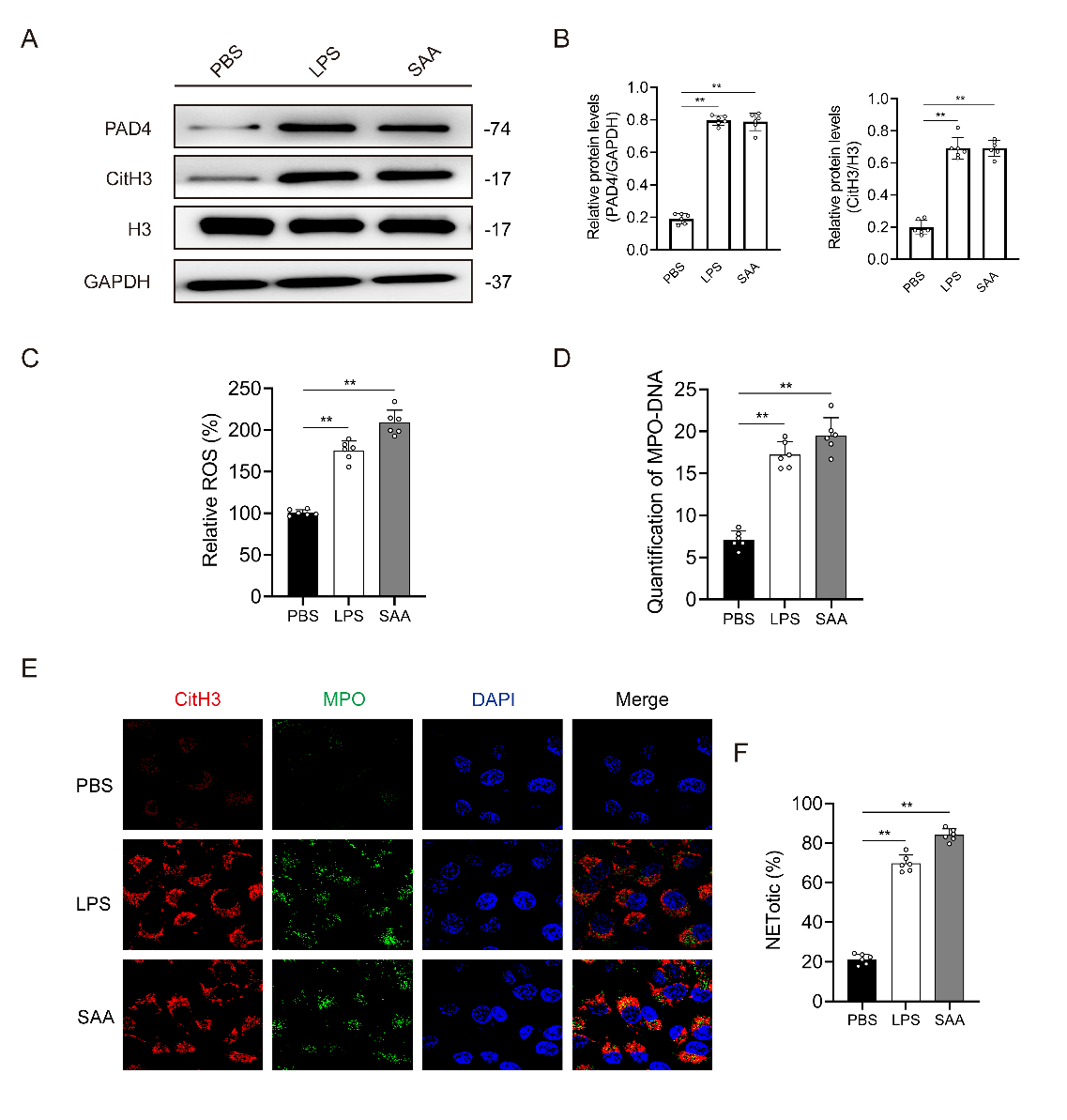
Supplementary Figure 6. SAA can promote NETs formation in vitro** A-B. analogous to the effect of lipopolysaccharide (LPS), stimulation by SAA can up-regulate the expression of PAD4 and CitH3 in neutrophils. Full-length blots are presented in Supplementary Figure 12; C-D. SAA can also induce the secretion of reactive oxygen species and MPO-DNA complex by neutrophils; E-F. the percent of NETotic neutrophil was significantly increased after SAA stimulation. For all subfigures: ** *P* < 0.01, the data are given as the mean ± SD. One-way ANOVA followed by the SNK-q post hoc test was used.


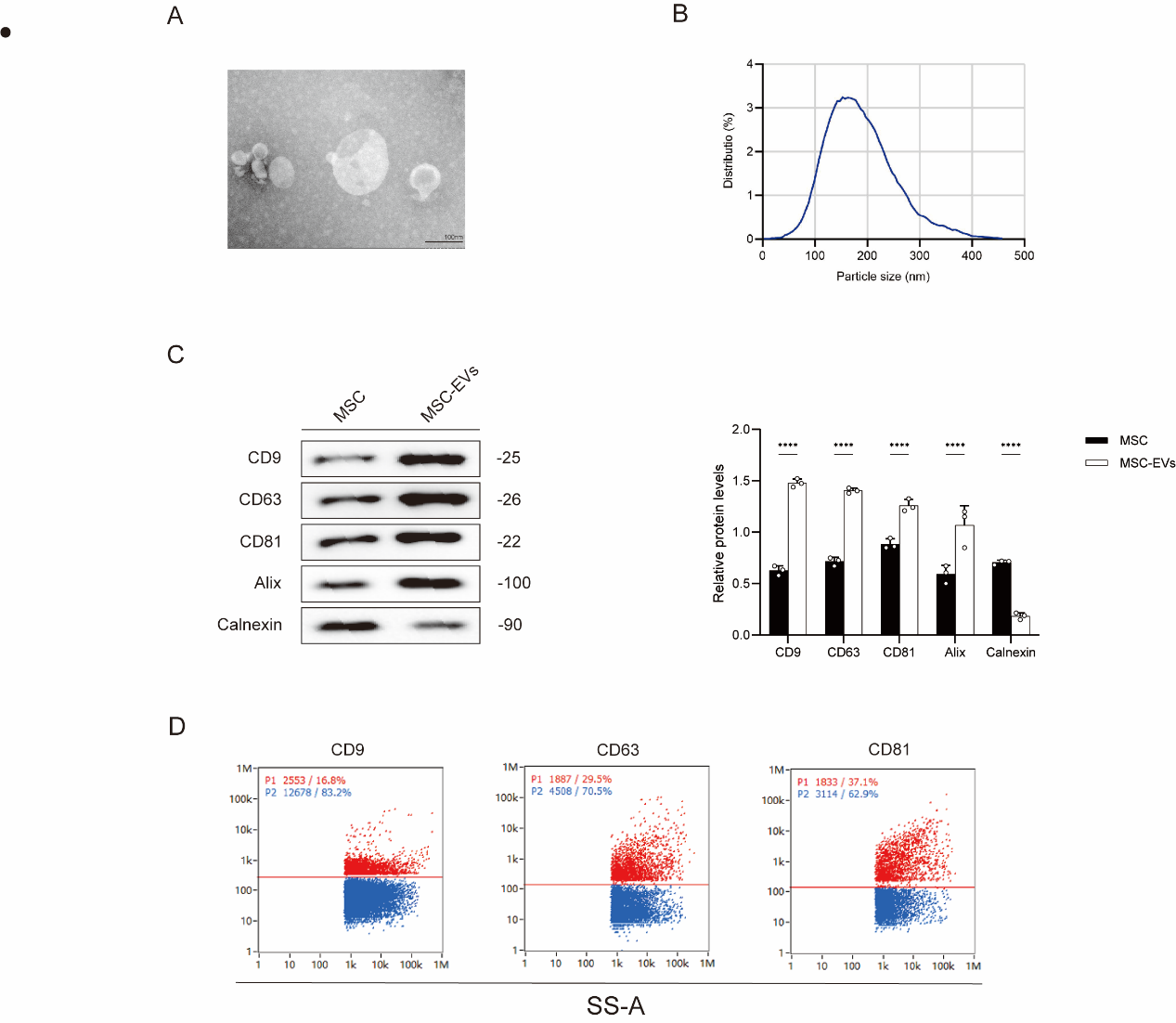


**Supplementary Fig. 7 Characterization of MSC-EVs.** A Representative transmission electron microscopic images of MSC-EVs. Scale bar = 100 nm. B DLS analysis reveals the particle distribution of exosomes in various sizes. C Western blot for the biomarkers of EVs, including CD9, CD63, CD81, and Alix. Calnexin was used as a negative control. Full-length blots are presented in Supplementary Figure 11 (D) .The expression of CD9 ,CD63 and CD81 detected by Flow Cytometry.***P< 0.001


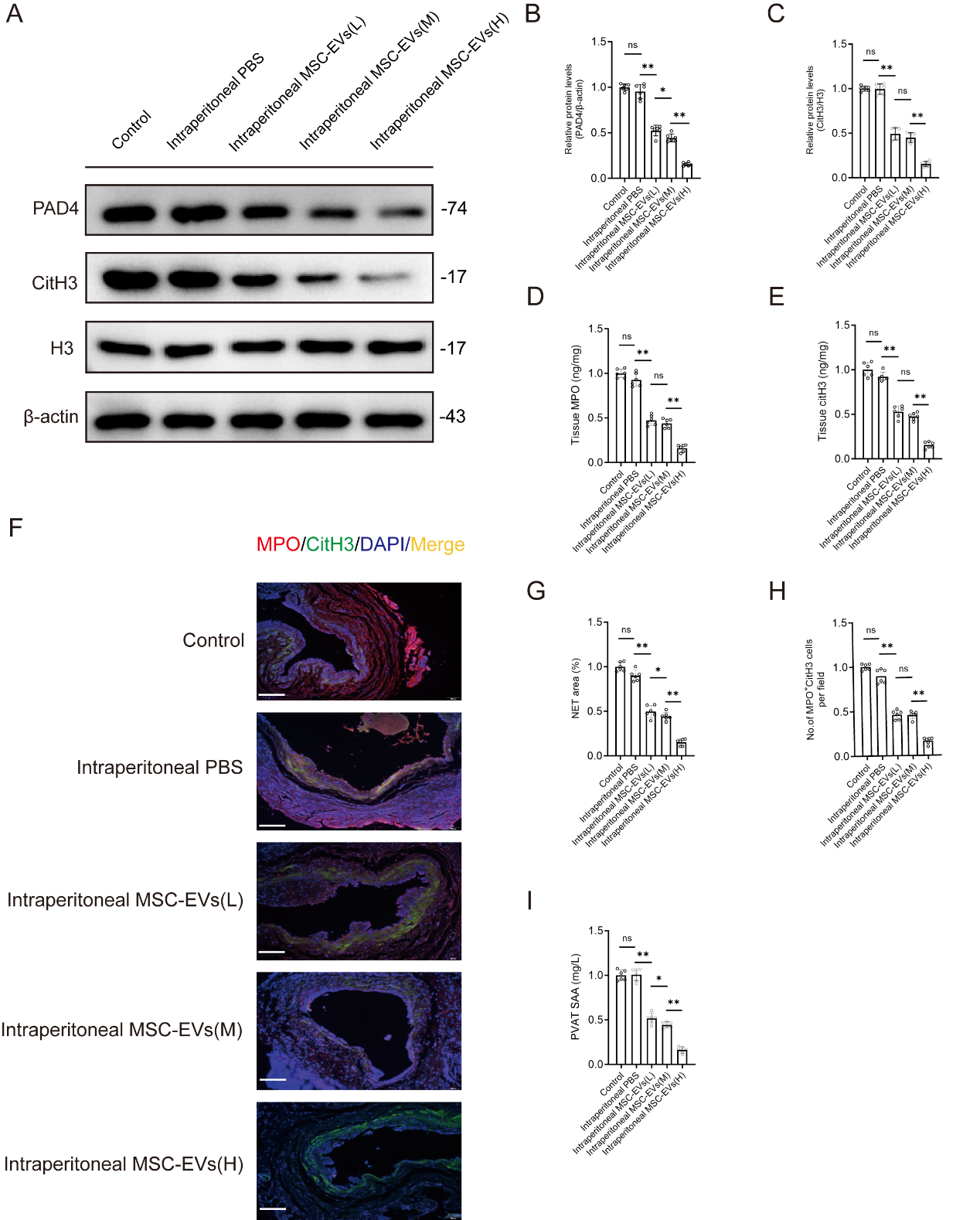


**Supplementary Figure 8. Intraperitoneal injection of MSC-EVs inhibits NET formation and SAA expression in a dose-dependent manner in the abdominal aorta.**

A-C**.** Intraperitoneal injection of MSC-EVs can suppress the expression of PAD4 and CitH3 in a dose-dependent manner in the abdominal aorta of mice receiving Ang II infusion. N = 6, one-way ANOVA followed by the SNK-q post hoc test. Full-length blots are presented in Supplementary Figure 13. **D-E.** The levels of CitH3 and MPO in the abdominal aorta of Ang II-infused mice were down-regulated following intraperitoneal injection of MSC-EVs in a dose-dependent manner. N = 6, one-way ANOVA followed by the SNK-q post hoc test. **F.** Immunofluorescence images showed that the expression of CitH3 and MPO in the aortic media layer of Ang II-infused mice was reduced after intraperitoneal injection of MSC-EVs in a dose-dependent manner. scale bar = 100μm. **G-H.** The number of MPO^+^CitH3^+^ neutrophils and the proportion of neutrophils undergoing NETosis were significantly decreased in Ang II-infused mice after intraperitoneal injection of MSC-EVs in a dose-dependent manner. N = 6, one-way ANOVA followed by the SNK-q post hoc test. **I.** SAA expression in PVAT in Ang II-infused mice was suppressed by intraperitoneal injection of MSC-EVs in a dose-dependent manner. N = 6, one-way ANOVA followed by the SNK-q post hoc test. For all subfigures: NS, not significant; ** *P* < 0.01. Data are presented as mean ± SD.

**References**

1. Foster MC, Hwang S-J, Porter SA, Massaro JM, Hoffmann U, Fox CS. Development and reproducibility of a computed tomography-based measurement of renal sinus fat. *BMC Nephrol* 2011;**12**:52.

2. Shields KJ, Stolz D, Watkins SC, Ahearn JM. Complement proteins C3 and C4 bind to collagen and elastin in the vascular wall: a potential role in vascular stiffness and atherosclerosis. *Clin Transl Sci* 2011;**4**:146-152.

3. Zagorchev L, Oses P, Zhuang ZW, Moodie K, Mulligan-Kehoe MJ, Simons M, Couffinhal T. Micro computed tomography for vascular exploration. *J Angiogenes Res* 2010;**2**:7.

**Original western blots**

**Supplementary Figure 9. Full-length blots of Figure 4A**


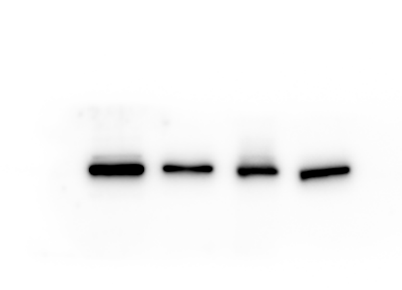


PAD4 -74KDa


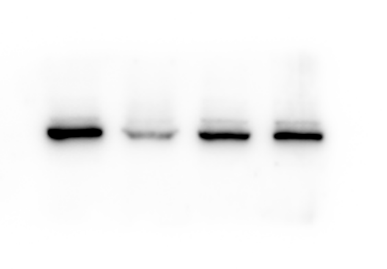


CitH3 -17KDa


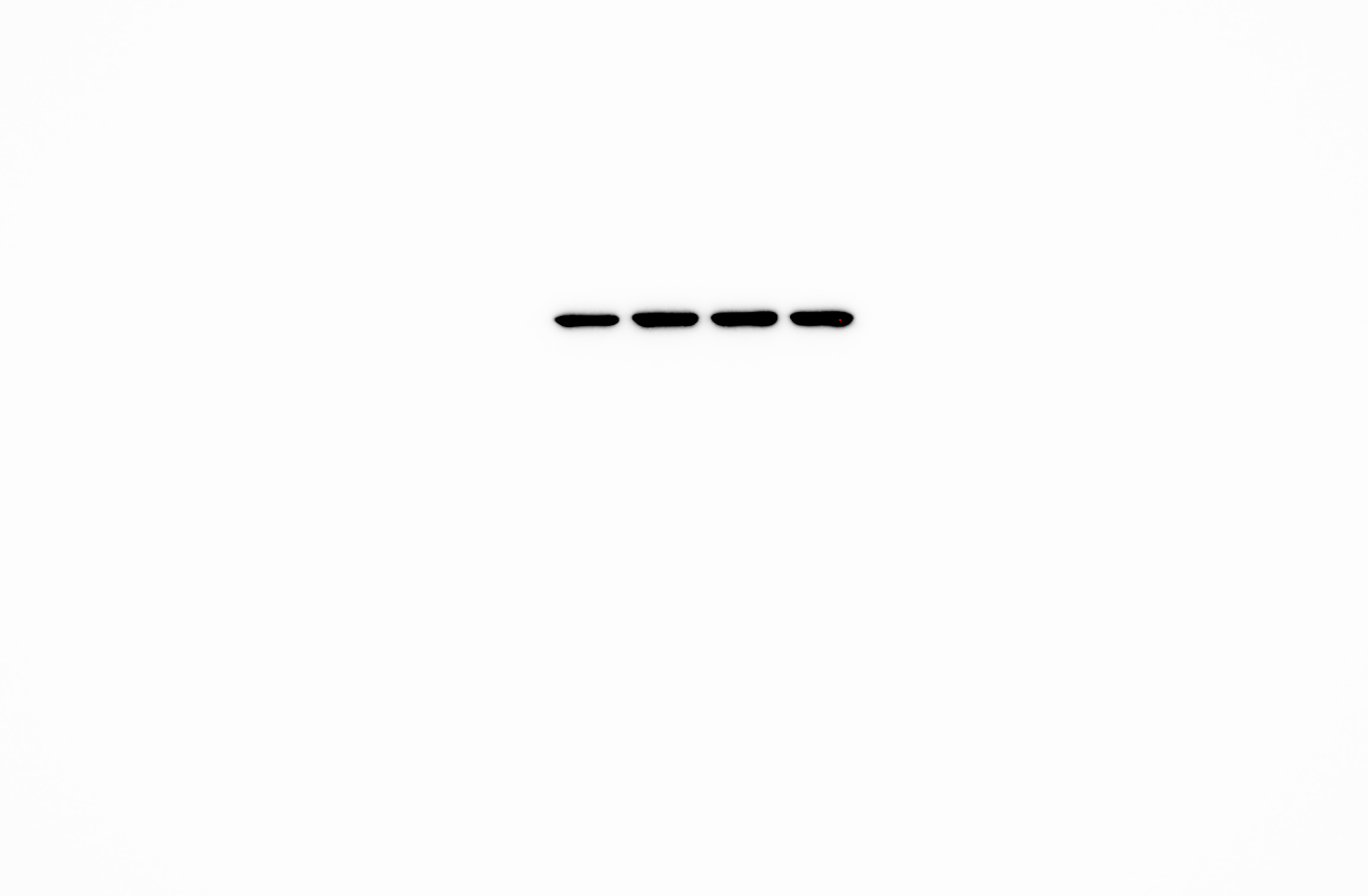


H3 -17KDa


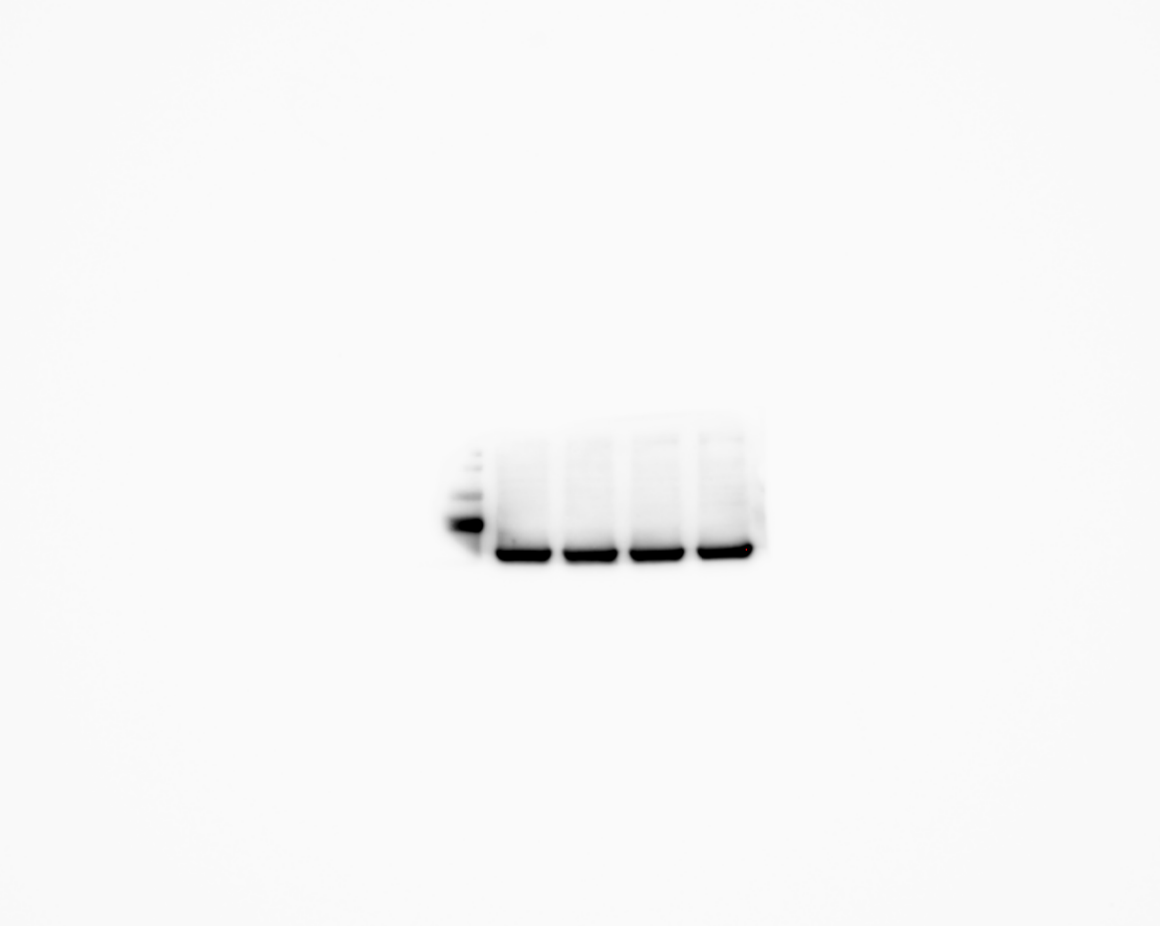


β-actin -43KDa

**Supplementary Figure 10. Full-length blots of Figure 6A**





PAD4 -74KDa


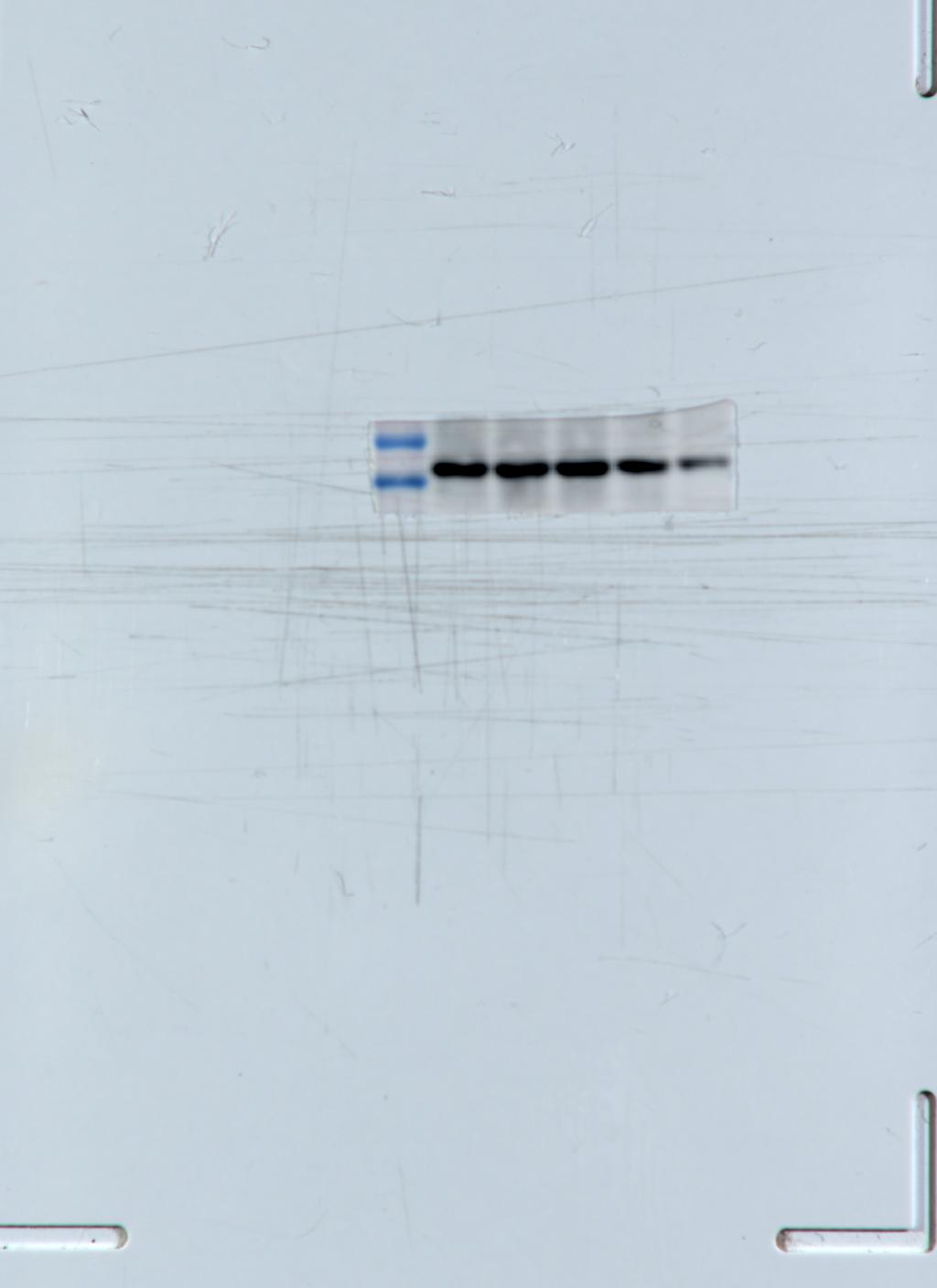


CitH3 -17KDa


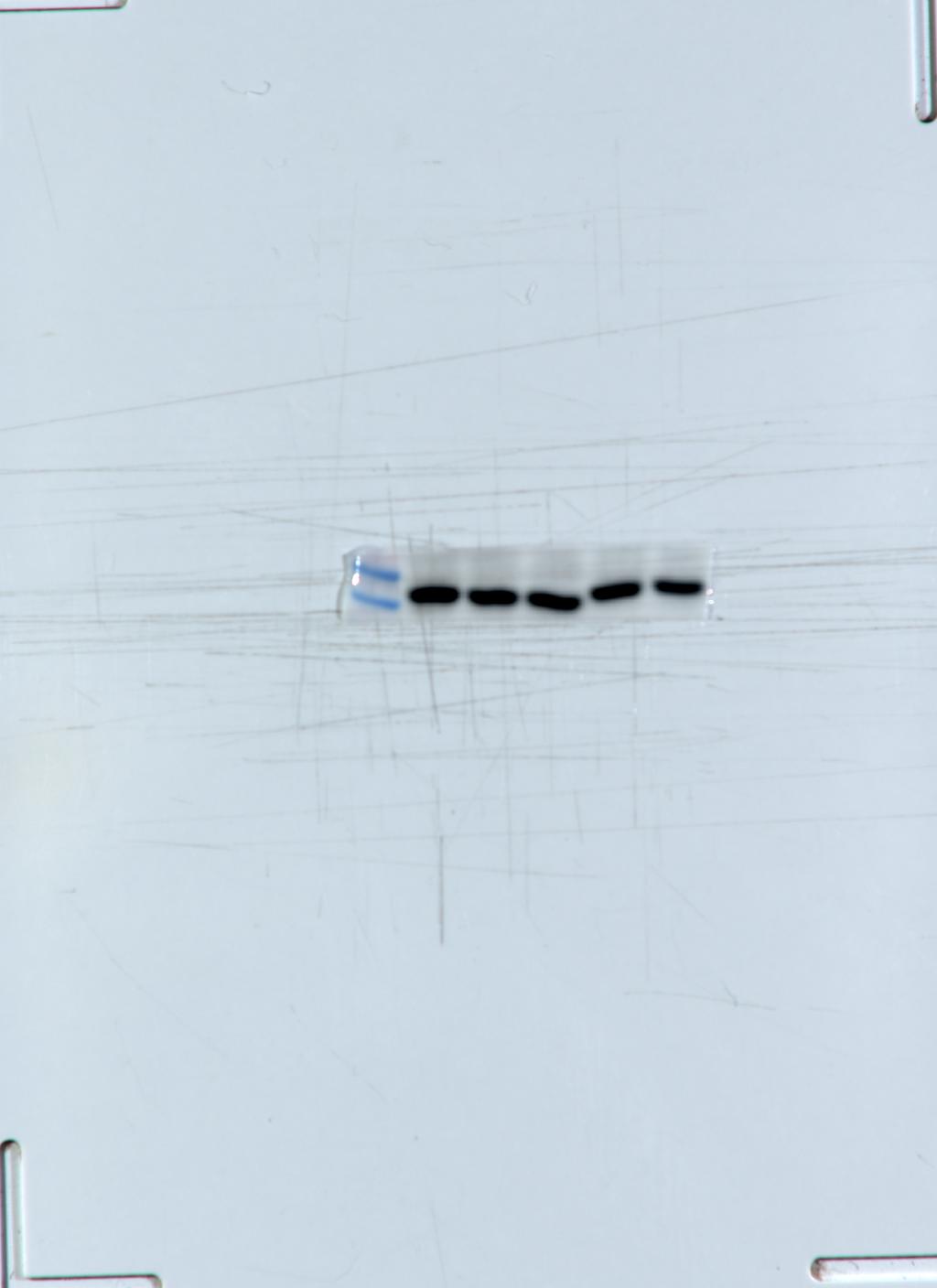
H3 -17KDa


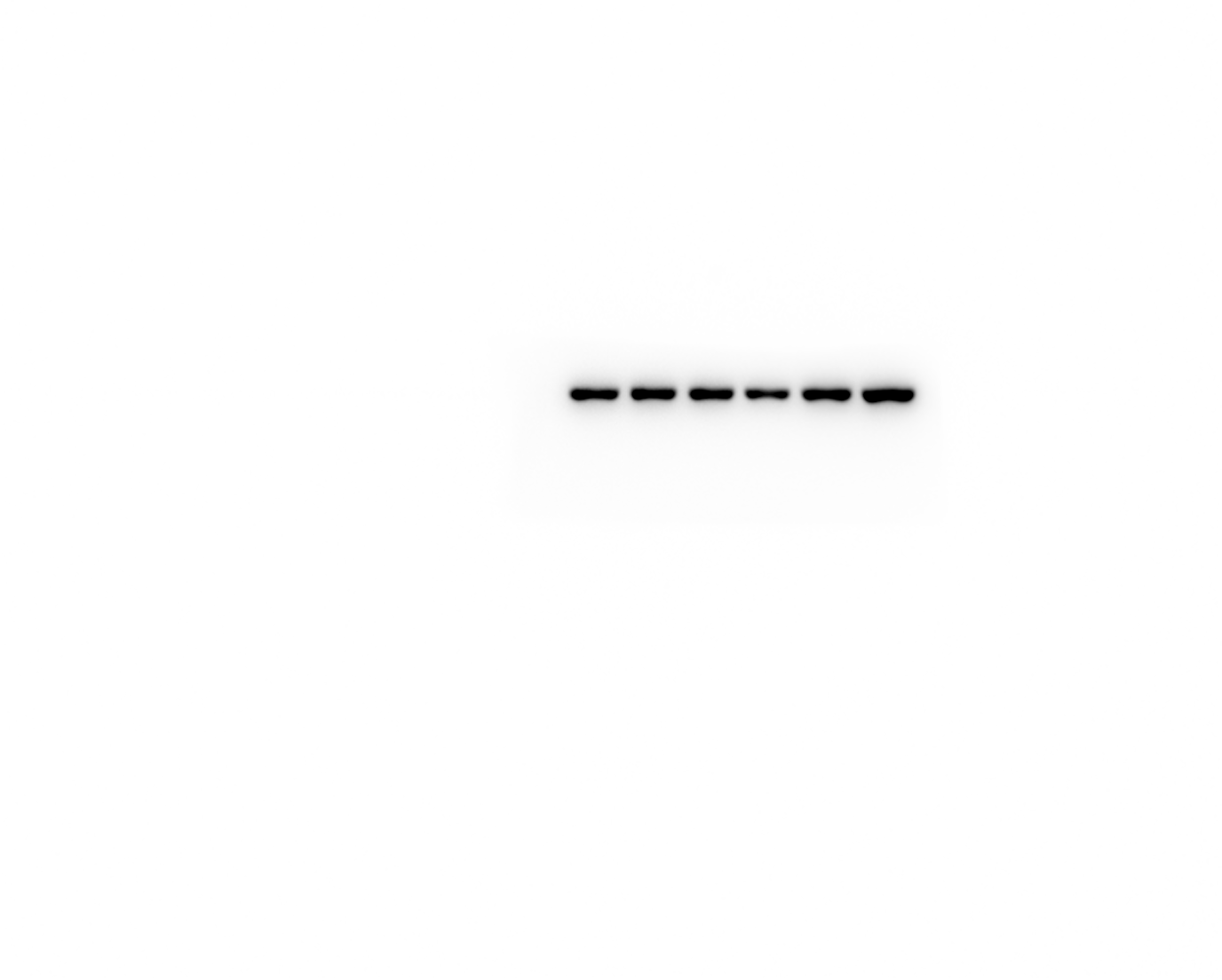


β-actin -42KDa

**Supplementary Figure 11. Full-length blots of Supplementary Figure 6A**





PAD4 -74KDa





CitH3 -17KDa


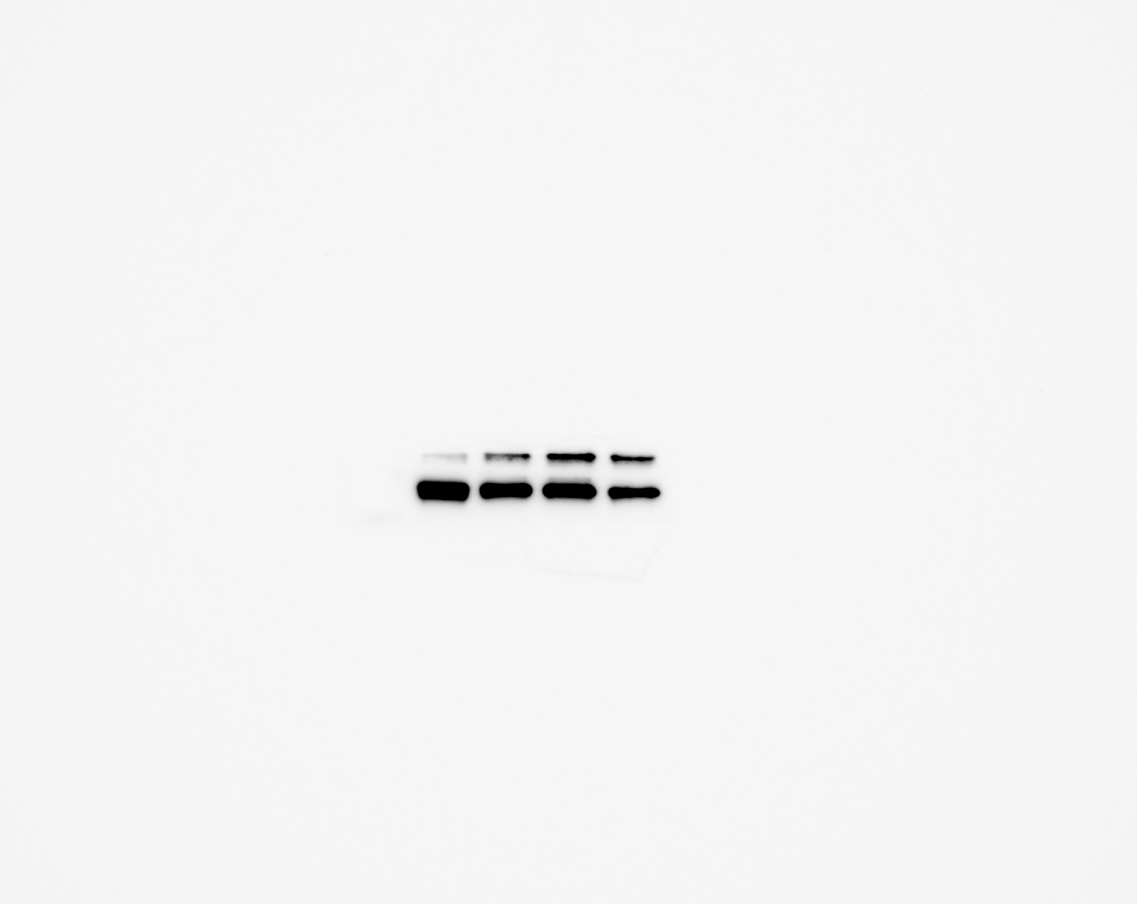


H3 -17KDa


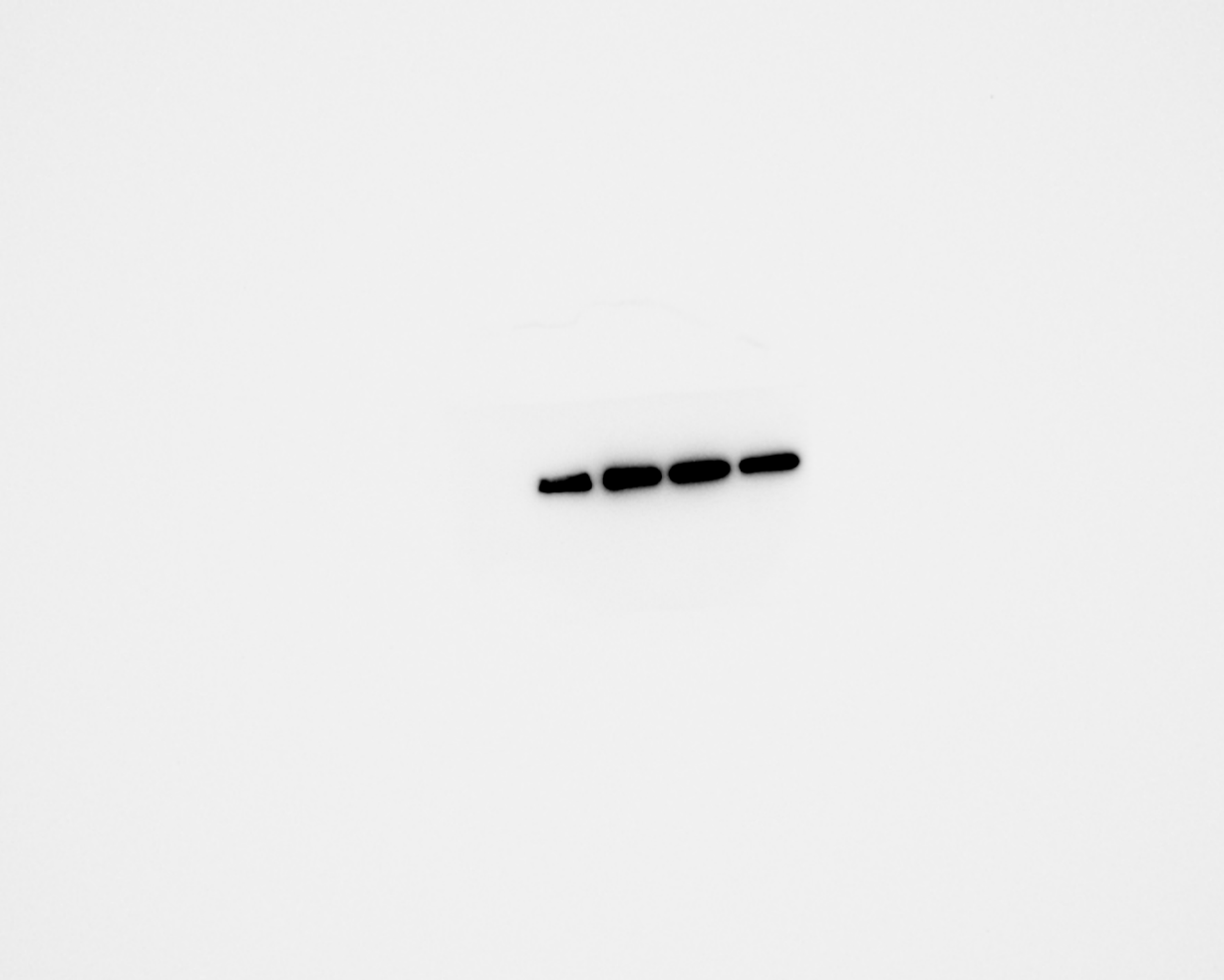


GAPDH -37KDa

**Supplementary Figure 12. Full-length blots of** **Supplementary Figure 7C**


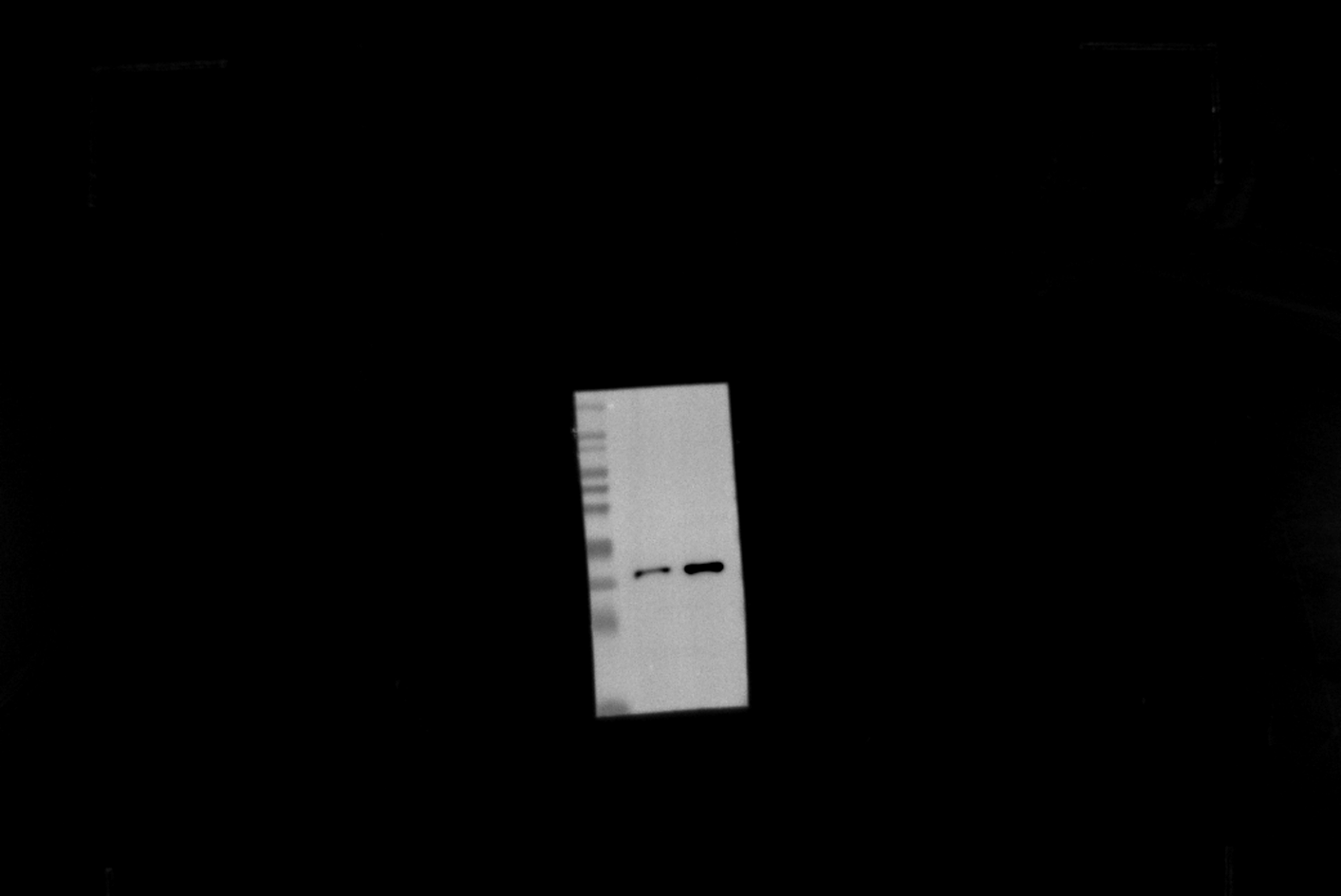


CD9 -25KDa


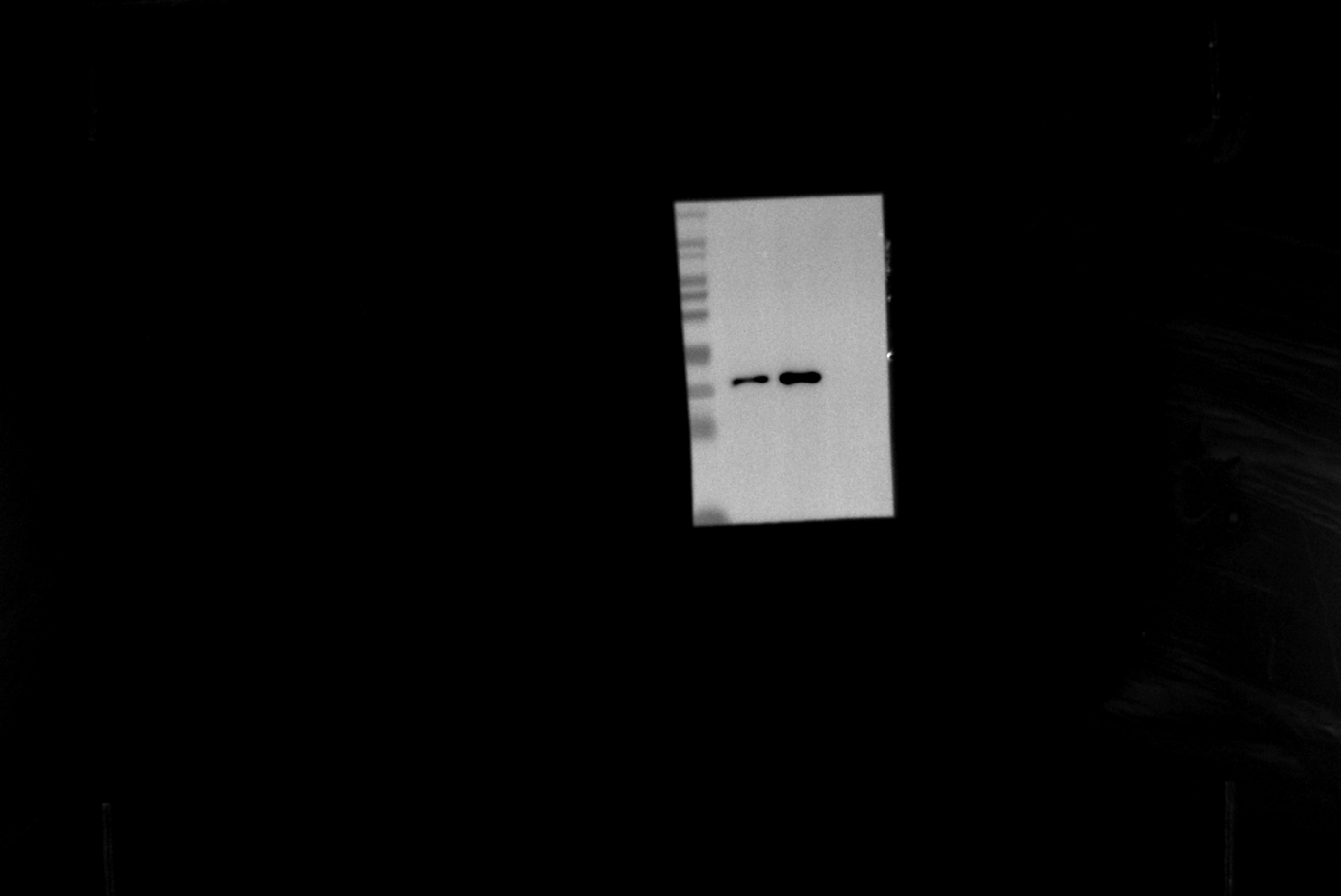


CD63 -26KDa


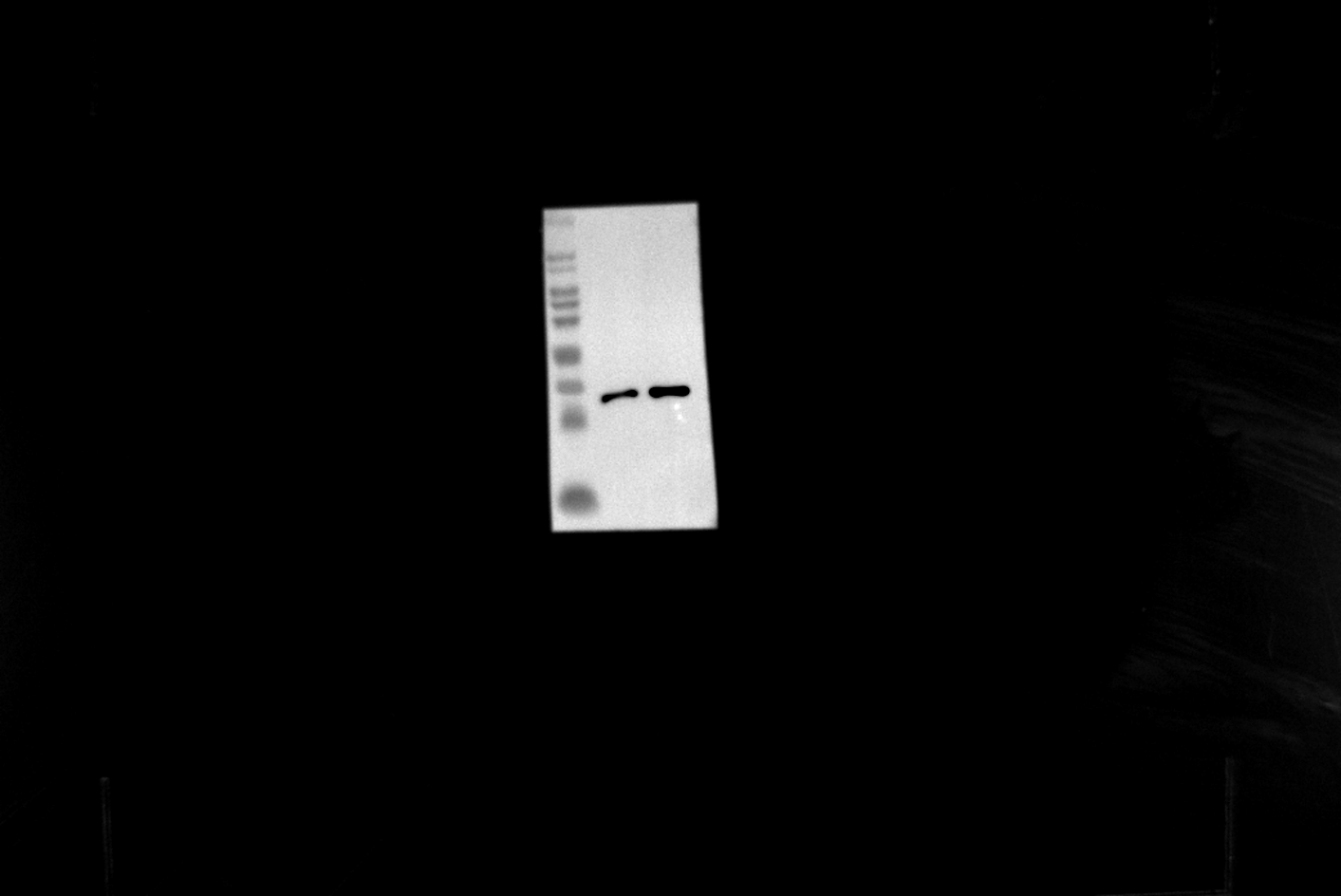


CD81 -22KDa


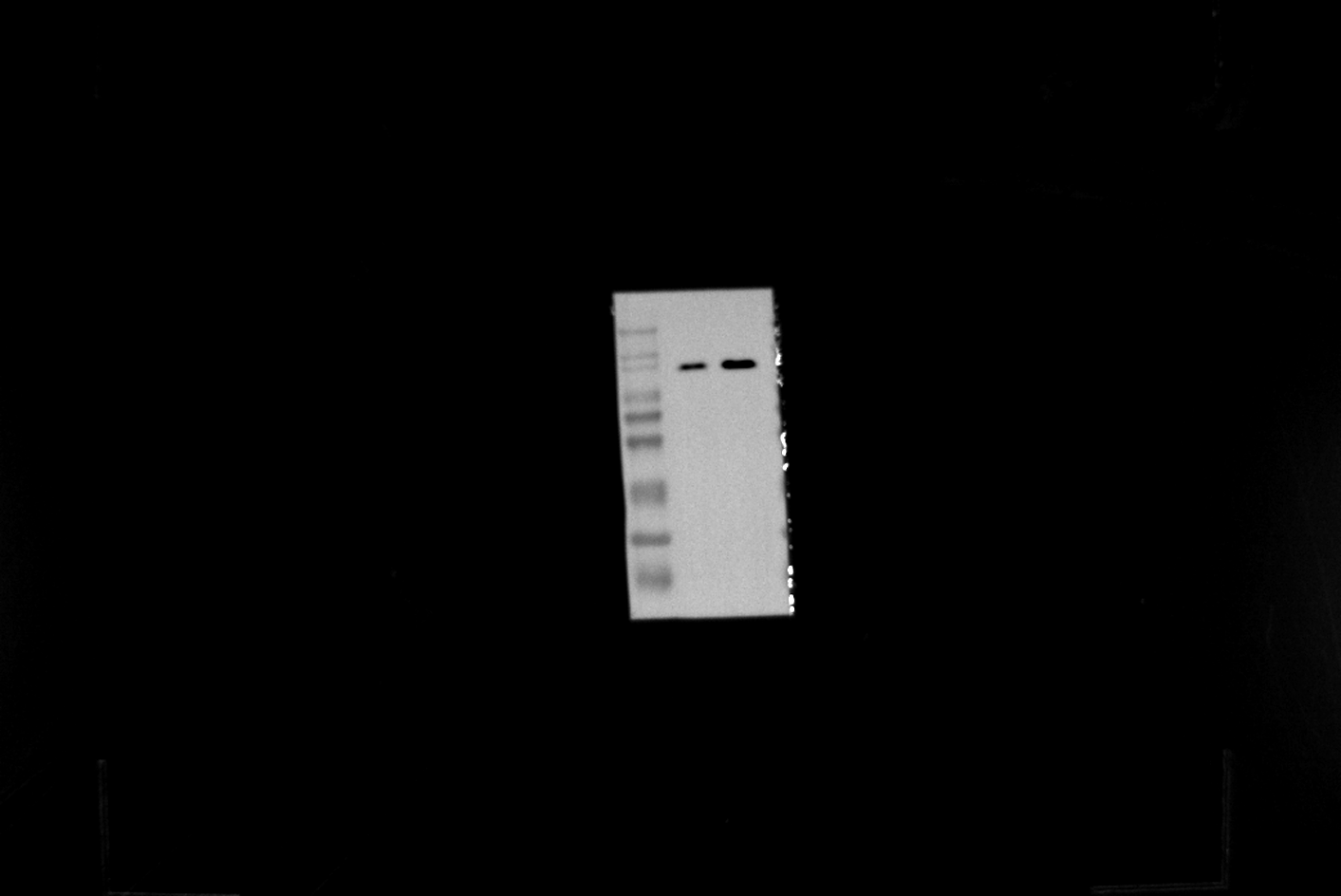


Alix -100KDa


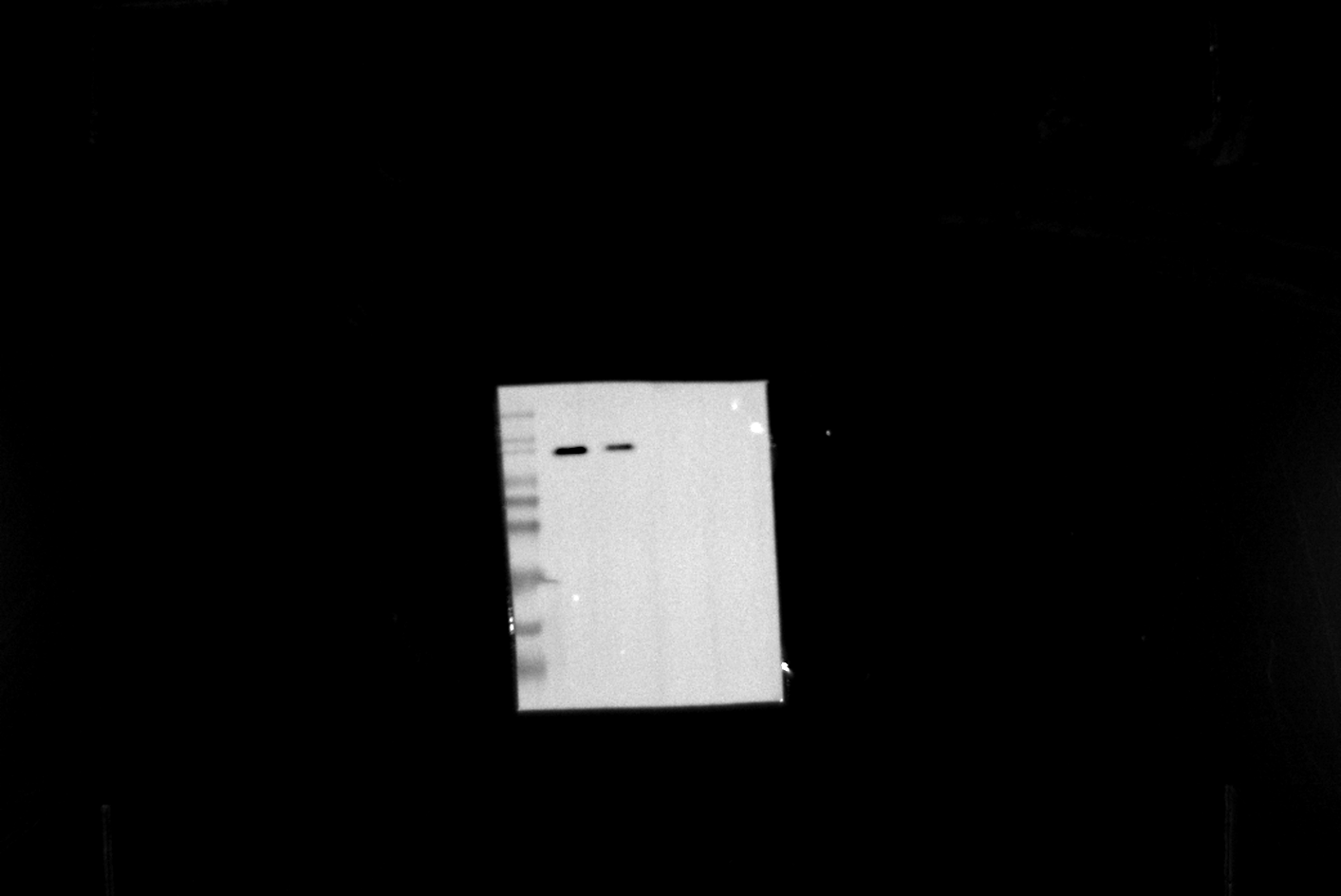


Calnexin -90KDa

**Supplementary Figure 13. Full-length blots of Supplementary Figure 8A**


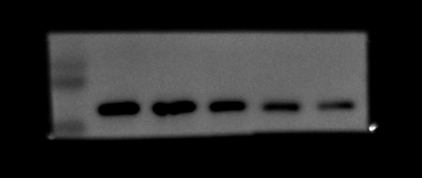


PAD4 -74KDa


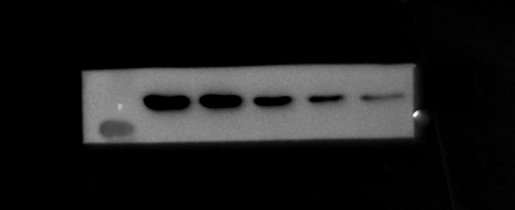


CitH3 -17KDa


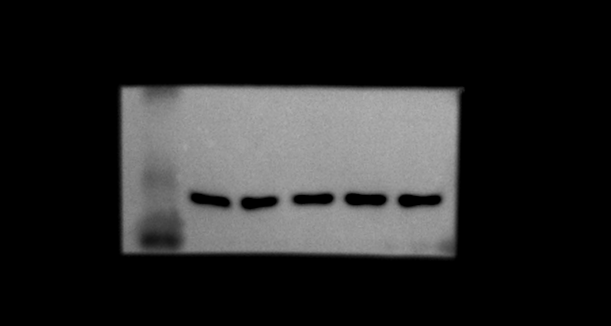
H3 -17KDa


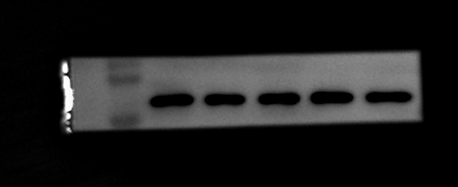


β-actin -42KDa
